# EHA Guidelines on Management of Antithrombotic Treatments in Thrombocytopenic Patients With Cancer

**DOI:** 10.1097/HS9.0000000000000750

**Published:** 2022-07-13

**Authors:** Anna Falanga, Avi Leader, Chiara Ambaglio, Zsuzsa Bagoly, Giancarlo Castaman, Ismail Elalamy, Ramon Lecumberri, Alexander Niessner, Ingrid Pabinger, Sebastian Szmit, Alice Trinchero, Hugo Ten Cate, Bianca Rocca

**Affiliations:** 1 University of Milano Bicocca, School of Medicine, Monza, Italy; 2 Department of Transfusion Medicine and Hematology, Hospital Papa Giovanni XXIII, Bergamo, Italy; 3 Institute of Hematology, Davidoff Cancer Center, Rabin Medical Center, Petah Tikva, Israel; 4 Sackler School of Medicine, Tel Aviv University, Tel Aviv, Israel; 5 Faculty of Medicine, Department of Laboratory Medicine, Division of Clinical Laboratory Sciences, University of Debrecen, Hungary; 6 ELKH-DE Cerebrovascular and Neurodegenerative Research Group, Debrecen, Hungary; 7 Center for Bleeding Disorders and Coagulation, Careggi University Hospital, Florence, Italy; 8 Hematology and Thrombosis Center, Hôpital Tenon, Hôpitaux Universitaires de l’Est Parisien, Assistance Publique Hôpitaux de Paris, Faculté de Médecine, Sorbonne Université, Paris, France.; 9 Research Group “Cancer, Haemostasis and Angiogenesis,” INSERM U938, Centre de Recherche Saint-Antoine, Institut Universitaire de Cancérologie, Faculty of Medicine, Sorbonne University, Paris, France; 10 Hematology Service, Clínica Universidad de Navarra, Pamplona, Spain; 11 CIBERCV, Instituto de Salud Carlos III, Madrid, Spain; 12 Department of Internal Medicine II, Division of Cardiology, Medical University of Vienna, Austria; 13 Department of Medicine I, Clinical Division of Hematology and Hemostaseology, Medical University of Vienna, Austria; 14 Department of Pulmonary Circulation, Thromboembolic Diseases and Cardiology, Centre of Postgraduate Medical Education, Otwock, Poland; 15 Institute of Hematology and Transfusion Medicine, Warsaw, Poland; 16 Department of Medical Oncology and Haematology Clinic, University Hospital Zurich, Switzerland; 17 Department of Internal Medicine, Thrombosis Expertise Center, Maastricht University Medical Center and CARIM School for Cardiovascular Diseases, Maastricht, the Netherlands; 18 Center for Thrombosis and Haemostasis, Gutenberg University Medical Center, Mainz, Germany; 19 Department of Safety and Bioethics, Section of Pharmacology, Catholic University School of Medicine, Rome, Italy

## Abstract

In cancer patients, thrombocytopenia can result from bone marrow infiltration or from anticancer medications and represents an important limitation for the use of antithrombotic treatments, including anticoagulant, antiplatelet, and fibrinolytic agents. These drugs are often required for prevention or treatment of cancer-associated thrombosis or for cardioembolic prevention in atrial fibrillation in an increasingly older cancer population. Data indicate that cancer remains an independent risk factor for thrombosis even in case of thrombocytopenia, since mild-to-moderate thrombocytopenia does not protect against arterial or venous thrombosis. In addition, cancer patients are at increased risk of antithrombotic drug-associated bleeding, further complicated by thrombocytopenia and acquired hemostatic defects. Furthermore, some anticancer treatments are associated with increased thrombotic risk and may generate interactions affecting the effectiveness or safety of antithrombotic drugs. In this complex scenario, the European Hematology Association in collaboration with the European Society of Cardiology has produced this scientific document to provide a clinical practice guideline to help clinicians in the management of patients with cancer and thrombocytopenia. The Guidelines focus on adult patients with active cancer and a clear indication for anticoagulation, single or dual antiplatelet therapy, their combination, or reperfusion therapy, who have concurrent thrombocytopenia because of either malignancy or anticancer medications. The level of evidence and the strength of the recommendations were discussed according to a Delphi procedure and graded according to the Oxford Centre for Evidence-Based Medicine.

## INTRODUCTION

Thrombocytopenia (TP) exposes patients to bleeding complications and represents an important limiting trait for the use of antithrombotic treatments, including anticoagulant, antiplatelet, and fibrinolytic agents. In cancer patients, TP can result from bone marrow substitution/infiltration by the malignant process or, often, as a side effect of anticancer medications.

On the other hand, cancer patients often require antithrombotic treatments as malignancy is associated with an increased risk of both venous and arterial thrombosis.^1–3^ Moreover, contemporary anticancer therapy and supportive care allow for treatment of older patients with cardiovascular or cardioembolic comorbidities. Thus, cancer patients frequently have an indication for antithrombotic therapy before or after cancer diagnosis. However, TP, active cancer, and ongoing chemotherapy are routinely exclusion criteria in the major randomized controlled trials (RCT) on antithrombotic drugs for primary or secondary prevention of atherothrombotic or cardioembolic complications. Thus, the evidence for those patients relies on retrospective observational studies, small subgroups from RCT, registries, case series, or mechanism-based investigations.

In addition, cancer patients are at increased risk of antithrombotic-associated bleeding,^4,5^ further complicated by TP and acquired hemostatic defects.^6–8^ In the absence of antithrombotic treatment, the risk of major bleeding seems inversely related to the platelet count and appears to disproportionally increase at platelet values <25 × 10^9^/L, where the estimated rate is ~15%/y, based on a large registry of 3584 TP patients,^9^ as compared to the ~0.07%/y rate in a general and relatively healthy population.^10^ A post hoc analysis of a RCT testing different platelet transfusion thresholds in patients with hematological malignancy demonstrated an increase in WHO grade ≥2 bleeding at platelet counts <80 × 10^9^/L.^11^ However, there was no inverse relationship of increased bleeding with decreasing platelet count below this threshold, apart from patients undergoing autologous hematopoietic stem cell transplantation (HSCT) with platelet counts 1–5 × 10^9^/L.^11^ Similarly, other studies showed no clear inverse relationship between platelet counts between 10 and 50 × 10^9^/L and bleeding.^12,13^ However, data are conflicting.^6,14^ Additional factors that may contribute to bleeding risk in TP patients include fever, female sex,^14,15^ allogeneic HSCT, hematocrit ≤25%,^11^ age, severe liver disease and uremia, as detailed in the *General management of patients with antithrombotic therapy and TP* section.

Cancer patients remain at risk of venous and arterial thrombosis in spite of TP, since mild-to-moderate TP does not protect against arterial or venous thrombosis and is associated with more adverse outcomes.^16–24^ General risk factors (age, diabetes, obesity, tobacco use) and specific mechanisms (inflammation, hypercoagulability) are shared between cancer and thrombosis. Moreover, some chemotherapeutic agents may induce endothelial dysfunction.^25,26^ Finally, during the post-nadir, recovery phase, chemotherapy-induced TP is associated with an increased output of immature platelets, known to be hyper-reactive and associated with more major arterial events in nononcological patients.^27^ Thus, TP should be interpreted in its clinical, oncological, and pharmacological context.

In immune-mediated TP, the risk of a first serious vascular arterial event (myocardial infarction [MI], stroke) is ~1.5%/y^9^ higher than in the general population (<1%/y),^10^ and appears not associated with a specific platelet count threshold. Moreover, following an acute ischemic or bleeding event, overall mortality and cardiovascular (CV) mortality are up to 4- to 5-fold higher in TP patients than in the non-TP counterpart.^9,19^ Under-use of antiplatelet therapy (APT) because of TP, especially when associated with cancer,^20,23,28^ and of revascularization,^20,29^ likely contribute to high CV mortality rate, together with other comorbidities. Furthermore, TP patients with or without cancer have been shown to receive less athero- and vascular-protective drugs other than APT, such as statins or beta-blockers,^20^ which instead are shown to be effective in cancer patients with serious vascular events (SVE).^30^ Notably, the overall 1-year survival rate of cancer patients post acute coronary syndrome (ACS) is as low as 26%, independently of baseline TP level.^31^

Cancer is also associated with an increased venous thromboembolism (VTE) incidence as compared to noncancer population with a relative risk as high as 14.91 (95% confidence interval [CI], 8.90-24.95) in a recent cohort study, that demonstrated a VTE proportion of 5% among cancer patients compared to <1% in patients without cancer.^32^ These patients also show an increased risk of VTE recurrence during anticoagulant therapy as compared to the noncancer population. In a landmark study of patients treated with vitamin K antagonists (VKA) for VTE, the 6-month VTE recurrence rate in cancer was as high as 20.7% (95% CI, 15.6%-25.8%) versus 6.8% (95% CI, 3.9%-9.7%) in patients without cancer.^4^ The on-therapy VTE recurrence rate in cancer patients is lower with low-molecular weight heparin (LMWH) and direct oral anticoagulants (DOACs).^33–35^ Cancer patients receiving anticoagulation caused by VTE, are at increased risk of bleeding especially when platelet counts are <50 × 10^9^/L.^16,36^

The Scientific Working Group on Bleeding and Thrombosis of the European Haematology Association (EHA) prompted the development of these Guidelines to provide a clinical practice guidance to help clinicians in the management of patients with cancer and TP in need of antithrombotic treatments. The Task Force Members were selected by the EHA in collaboration with the European Society of Cardiology (ESC), to represent professionals involved in the medical care of patients with this disease combination.

Selected experts from both Societies undertook a comprehensive review of the published evidence for management of a given condition. A critical evaluation of diagnostic and therapeutic procedures was performed, including assessment of the risk-benefit ratio. The level of evidence and the strength of the recommendation of particular management options were weighed and graded according to the Oxford Centre for Evidence-Based Medicine,^37^ as outlined later.

## METHODOLOGY

### Target population

This EHA/ESC scientific document focuses on adult patients with active cancer and a clear indication for anticoagulation, single or dual APT, their combination, or reperfusion therapy, and who have concurrent TP caused by either malignancy or anticancer medications.

The anticipated duration of TP was considered to be days to weeks, unless otherwise specified. The guideline was adjusted for prolonged TP (ie, >4 wks) wherever relevant.

These guidelines exclude TP associated with disseminated intravascular coagulation,^38^ congenital bleeding disorders and antiphospholipid syndrome, as well as patients receiving palliative care since the risk-benefit ratio of antithrombotic treatment may be different in those contexts because of the underlying disorder or limited life expectancy.^39,40^

Moreover, these guidelines also do not address specific recommendations regarding the management of bleeding or rethrombosis, while on antithrombotic treatment, assuming that the context of TP would not require different actions as needed in non-TP patients.

### Recommendation process

The task force consisted of hematologists, cardiologists, thrombosis specialists, clinical pharmacologist, vascular medicine specialists, and a vascular neurologist, identified by EHA and ESC. The task force reviewed and graded the available evidence by performing a nonsystematic literature review of the pubmed database using the keywords listed in the Suppl. Appendix S1. The title and abstract of the candidate articles were screened. The reference lists of the relevant articles were screened for additional papers.

Recommendations were discussed and a final consensus was reached by the Delphi method (see Suppl. Appendix S2). The Oxford Centre for Evidence-Based Medicine Levels of Evidence were used to evaluate and classify the level and grade of evidence supporting each recommendation (**Table 1**).^37^

**Table 1.** Level and Grades for Evidence-based Recommendations

Level	Definition	Grade	Definition
1	SR (with homogeneity) of RCTs	A	Consistent level 1 studies
1b	Individual RCT (with narrow “confidence interval”)		
1c	All or none^a^		
2	SR (with homogeneity) of cohort studies	B	Consistent level 2 ***or***3w
2b	Individual cohort study (including low-quality RCT; eg, <80% follow-up)		
2c	“Outcomes” research; ecological studies		
3	SR (with homogeneity) of case-control studies		
3b	Individual case-control study		
4	Case series (and poor quality cohort and case-control studies)	C	Level 4 studies ***or*** extrapolations from level 2 or 3 studies
5	Expert opinion without explicit critical appraisal, or based on physiology, bench research or “first principles”	D	level 5 evidence ***or*** troublingly inconsistent or inconclusive studies of any level

^a^Met when all patients died before the treatment became available, but some now survive on it; or when some patients died before the treatment became available, but none now die on it.

RCT = randomized controlled trials; SR = systematic review.

Adapted from the Oxford Centre for Evidence-Based Medicine: Levels of Evidence.^37^

This document has undergone double-blinded peer review process in the HemaSphere journal and has been approved by the HemaSphere editors.

### Definitions

For the purpose of this document:

*TP* is defined as platelet counts ≤100 × 10^9^/L in association with cancer with or without active chemotherapy. Platelet count (× 10^9^/L) is stratified according to the National Cancer Institute Common Terminology Criteria for Adverse Events (NCI CTCAE)^41^ as shown in **Table 2**, with a minor modification: the upper limit of grade 1 was agreed to be 100 × 10^9^/L rather than the lower limit of the local reference range.
*Antithrombotic medication management* refers to any dose or type of antithrombotic medication including supportive care aiming to mitigate the thrombotic or bleeding risk, such as intravenous catheter removal or platelet transfusion.
To *Hold* antithrombotic medication means to withdraw the antithrombotic drug(s) (temporarily or permanently, as indicated) because of TP.
*Active cancer* is defined as one of the following: cancer diagnosed within the previous 6 months; recurrent, regionally advanced or metastatic cancer; cancer for which treatment had been administered within 6 months; or cancer that is not in complete remission.^42^ The guidelines include general recommendations relevant to all populations, as well as indication-specific subgroups.

**Table 2.** Grades of Thrombocytopenia

	Grade 1	Grade 2	Grade 3	Grade 4
Platelet range (× 10^9^/L)	<100 to 75	<75 to 50	<50 to 25	<25

The grades of thrombocytopenia are based upon National Cancer Institute Common Terminology Criteria for Adverse Events (NCI CTCAE).^41^ The only modification is that the upper limit of grade 1 will be 100 × 10^9^/L and not the lower limit of the local reference range.

## ANTITHROMBOTIC THERAPY

### General management of patients with antithrombotic therapy and TP

To provide evidence-based recommendations, both the thrombotic and bleeding risk of the individual patient should be carefully considered and balanced. A prevailing bleeding risk, in addition to the TP, should further support a decision to hold or reduce antithrombotic therapy. On the other hand, a prevailing thrombotic risk should drive toward continuing antithrombotic therapy at a full or reduced dose. **Tables 3 and 4** provide a consensus-based nonexhaustive list of thrombotic and bleeding risk factors, respectively, for cancer patients with an indication for antithrombotic treatment. It is worth noting that some parameters lack formal validation on large, prospective cohorts.

**Table 3.** Factors That Increase the Risk of Thrombosis

Underlying Thrombotic Disorder or Risk Factor	Thrombosis Risk Level	
	Very-high Risk^a^	High Risk^b^
ASCVD	Previous ACS Stable angina, Coronary revascularization (PCI, CABG, and other), Stroke and TIA PAD Documented plaques on coronary angiography or CT scan, or on carotid ultrasound; DM with target organ damage, or at least 3 major risk factors, Early onset of T1DM of long duration (> 20 y); Severe CKD (eGFR < 30 ml/min/1.73 m^2^)	
Left ventricular thrombus	Estimated MACE 37.1%; mortality 18.9%; stroke 13.3%; over 2 y	
Mechanical heart valves	Risk of ischemic stroke >10%/y: mitral position, recently placed prosthesis (<3 mo), prosthesis and additional CV risk factor, specific types of valve (Starr Edwards, Bjork Shiley); aortic position with additional risk factors (AF, LVEF <35%, history of thromboembolism)	
Mitral biological valve		Systemic TE >5%/y at <3 mo after implantation
Aortic biological valve		Systemic TE >5%/y at <3 mo after implantation plus recent history of TE or presence of left atrial thrombi
Rheumatic mitral valve disease		- plus AF - plus left atrial diameter >55 mm or left atrial thrombus or prior TE
AF	Plus recent (<30 d) cardioembolic stroke 10.1% of recurrent ischemic stroke, TIA, and systemic arterial embolism	CHA_2_DS_2_-VASc score ≥4 Risk of ischemic stroke ≥4.8%/y Risk of ischemic stroke/TIA/systemic embolism ≥6.7%/y
AF + ASCVD	AF plus recent PCI <30 d	AF and PCI/ACS in the previous 12 mo
PFO/LAA closure		Thrombus formation on the device: ~1% of patients undergoing ASD/PFO closure and 2–5% of patients undergoing LAA closure
PE/DVT	PE with hemodynamic instability	- Acute DVT - PE since diagnosis <6 wks
Type of cancer		- >4%/y: brain tumors, multiple myeloma, pancreas, stomach cancer (as advanced/metastatic disease) - Khorana score ≥2
Chemotherapy/anticancer therapy		- Venous TE: • Cisplatin-based chemotherapy • Cytostatic: capecitabine, gemcitabine, paclitaxel • Tamoxifen • Immunomodulatory drugs: thalidomide, lenalidomide - Arterial TE: • Aromatase inhibitors • Androgen-deprivation therapy • VEGF-targeted drugs (eg, bevacizumab, *ramucirumab*, *sunitinib*, *sorafenib*, *pazopanib*, *axitinib*, *cabozantinib*, *regorafenib*, *lenvatinib*, *vandetanib*, *aflibercept*) • BCR-ABL TKI (nilotinib, ponatinib)
Cancer-associated conditions or comorbidities		- Cancer-related surgery - Allogeneic transplantation (subgroup with prior VTE) - APS

^a^Risk of fatal cardiovascular disease ≥1%/y.

^b^Risk of fatal cardiovascular disease: 0.5–<1%/y.

ACS = acute coronary syndrome; AF = atrial fibrillation; APS = antiphospholipid syndrome; ASCVD = Atherosclerotic cardiovascular disease; CABG = coronary artery bypass graft; CAD = coronary artery disease; CKD = chronic kidney disease; CT = computed tomography; CV = cardiovascular; DAPT = dual antiplatelet therapy; DM = diabetes mellitus; DVT = deep vein thrombosis; LAA = left atrial appendage; LVEF = left ventricular ejection fraction; MACE = major adverse cardiac events; PAD = peripheral artery disease; PCI = postpercutaneous coronary intervention; PE = pulmonary embolism; PFO = patent foramen ovale; TIA = transient ischemic attack; T1DM = diabetes mellitus type 1; TE = thromboembolism; VEGF = vascular endothelial growth factor; VTE = venous thromboembolism.

**Table 4.** Bleeding Risk Factors in Cancer Patients With TP

Risk Factor	Population Characteristics in Supporting Studies			30-d Risk of Major Bleeding^a^	Ref
	Cancer Type	TP	AT Rx		
Independent risk factors (not specific to cancer and thrombocytopenia)					
Recent major bleeding^b^	General	Yes	Yes	High	^43,44^
Age <18 y	General	Yes	No	High	^45^
Age >60 y	General	No	Yes	Low-Int	^46^
CKD ≥stage III	General	Yes	Yes	High	^44^
	General	No	Yes		^47^
Hypertension	General	Yes	Yes	Low-Int	^44^
BMI ≥ 40	General	No	Yes	Low-Int	^46^
Disease-related factors					
Acute leukemia	AML, APL	Yes	No	High	^14,48,49^
Unresected primary tumor	GI, genitourinary, gynecologic	No	Yes	Low-Int	^46,47^
Bone marrow involvement	General	Yes	No	High	^50^
		No	Yes		^47^
Primary or metastatic brain cancer	General	No	Yes	High	^51^
Fever^c^	General	Yes	No	Low-Int	^50^
		No	Yes		^44^
Anemia	Hematological cancers	Yes	No	Low-Int	^15^
	General	No	Yes		^46^
DIC	APL	Yes	No	High	^49^
Poor performance status	Cancer ^t^ (n=609)	Yes	No	Low-Int	^50^
Treatment-related factors					
HSCT	Allogeneic	No	Yes	High	^44,52,53^
		Yes	No		^11,13,18^
	Autologous	Yes	No	Low-Int	^11^
Graft versus host disease	Allogeneic HSCT	No	Yes	Low-Int	^18^
Platinum-based regimens	General	Yes	No	Low-Int	^50,54^
Taxane- or gemcitabine-based regimens	General	Yes	No	Low-Int	^54,55^

This table shows risk factors for major bleeding, based on evidence from cancer patients with either TP or antithrombotic therapy (or both). The table includes factors associated with an increased relative risk of bleeding, stratified into low-intermediate (shaded orange) and high (shaded dark red) based on absolute 30-d major bleeding rates.^a^

^a^Defined as 30-d major bleeding risk in studies of cancer patients with TP: low-intermediate 0–5%; high ≥ 6%.

^b^Defined as major bleeding in the past 4 wks.

^c^Including fever, febrile neutropenia, infection, sepsis.

AML = acute myeloid leukemia; APL = acute promyelocytic leukemia; AT Rx = antithrombotic therapy; BMI = body-mass index; CKD = chronic kidney disease; DIC = disseminated intravascular coagulopathy; GI = gastrointestinal; HSCT = hematopoietic stem cell transplantation; Low-Int = low-intermediate; RBC = red blood cell; TP = thrombocytopenia.

Furthermore, clinically relevant drug-drug interactions (DDIs) between some antithrombotic and anticancer drugs may further impact on the risk/benefit balance of either antithrombotic or anticancer drugs as reported in Suppl. Table S1 that provides a nonexhaustive list of clinically relevant DDIs.

We advise the following general approach toward all antithrombotic regimens in cancer patients with TP:

- To reassess the indication of the antithrombotic therapy, irrespective of TP.
- To assess the ongoing associated thrombotic and bleeding risks by identifying generic and cancer-specific factors (**Tables 3 and 4**).
- To anticipate the duration of grade 3–4 TP.
- To formulate a clear antithrombotic therapy management plan, to be reassessed frequently according to the individual treatment plan, kinetics of TP and possible complications or comorbidities.
- To consider restarting antithrombotic therapy, once the platelet count is consistently above a threshold deemed suitable for full antithrombotic medication, as indicated in each section.

Additional preventive strategies should be considered to minimize the bleeding risk associated with antithrombotic therapy in patients with cancer and TP. In particular, traditional nonsteroidal anti-inflammatory drugs (NSAIDs), often used as analgesics or antipyretics in cancer patients, are known to increase upper gastrointestinal (GI) bleeding by inhibiting cyclooxygenase (COX)-1-dependent gastric mucosal protection,^56^ whereas selective inhibitors of COX-2 have been associated with less GI bleeding as compared to traditional NSAIDs in RCTs.^57^ Moreover, in patients on single or combined antithrombotic drug(s), the risk of upper GI bleeding can be significantly lowered (by approximately 50%) by proton pump inhibitors (PPI).^58^ Thus, for TP cancer patients the use of a PPI and avoidance of traditional NSAIDs as analgesics or antipyretics, are central to preventing GI bleeding, optimizing the benefit/risk balance of antithrombotic therapy, as also recommended in recent guidelines.^59^ DDIs between clopidogrel and omeprazole have led regulatory agencies to discourage this combination, preferring other PPIs (eg, pantoprazole).^60^

Routine platelet function monitoring is not recommended in the general population, and, in the setting of TP, the results of the available tests could be unreliable.

**Recommendations**

**1. General recommendations for all antithrombotic regimens in cancer patients with TP**

**a.** In all patients on single or combined antithrombotic drugs, we advise against the use of traditional NSAIDs and high doses of aspirin (≥300 mg) as analgesic or antipyretic drugs. ***Level 1, grade A***
**b.** In all patients on single or combined antithrombotic drugs, we recommend using PPIs to prevent GI bleeding. ***Level 1, grade A***
**c.** Among patients receiving clopidogrel, omeprazole and esomeprazole are not recommended, and pantoprazole must be considered instead. ***Level 1, grade A***
**d.** Clinically relevant DDIs should be always considered, especially for clopidogrel, ticagrelor, warfarin, and dabigatran (see Suppl. Table S1). ***Level 1, grade D***
**e.** In all patients at high/very-high CV risk, we advise to always optimize the treatment of modifiable CV or cardioembolic risk factors including hypertension and hypercholesterolemia. ***Level 5, grade D***
f. Platelet function monitoring is not recommended to guide single or dual APT. ***Level 1, grade*A**

### Assessing the risk of TP in patients receiving antithrombotic therapy

Patients with hematological malignancies and patients receiving regimens based on platinum, gemcitabine, and anthracyclines have a ≥10% 3-month incidence of grade 3–4 TP,^61^ likely underestimated especially in patients with solid malignancy, since platelet counts are routinely measured before the next chemotherapy cycle in clinical practice and trials, rather than at the anticipated platelet’s nadir. Although several days of grade 3 TP between treatment cycles would not be clinically meaningful in most patients and remain undetected, this would expose patients receiving therapeutic-dose anticoagulation to a very-high risk of bleeding. In patients receiving anticancer regimens associated with a ≥10% incidence of grade 3–4 TP at 3–6 months, proactive measurement of platelet counts at the time of the anticipated nadir of TP would help to optimize antithrombotic management. If grade 3–4 TP is identified in patients receiving antithrombotic therapy, this could affect management, as detailed in the following sections of this document. Although grade 1–2 TP would usually not necessitate changes in antithrombotic therapy, it may not represent the true platelet nadir which could occur several days later. Therefore, in case of grade 1–2 TP, we recommend that the platelet count be rechecked within 1–2 days, to test for grade 3–4 TP.

This approach, advising patients based on test results, would increase healthcare burden to measure platelet counts. Although there is no supporting evidence, nevertheless we favor this approach, considering the absolute small patient number and the potential of preventing iatrogenic bleeding events, which would have higher impact on patient’s life and health care costs.

**Recommendations**

**2. Monitoring platelet counts in outpatients receiving antithrombotic and anticancer therapy**

**a.** For patients receiving antithrombotic drugs who have a moderate-high risk of developing grade 3–4 TP because of active anticancer therapy, we recommend measuring platelet count near the anticipated platelet count nadir. ***Level 5, grade D***
**b.** If no TP is identified within the first 3 chemotherapy cycles, we recommend against further monitoring. ***Level 5, grade D***
**c.** For grade 1–2 TP, platelet count should be rechecked within 1–2 days. ***Level 5, grade D***
**d.** For grade 3–4 TP, antithrombotic management should be managed according to specific recommendations. ***Level 5, grade D***

### Strategies for increasing platelet count

Increasing the platelet count in grade 3–4 TP may allow antithrombotic therapy in selected patients with a high-thrombotic risk whose TP would otherwise exclude such therapy.

Strategies for increasing platelet count may include platelet transfusion and use of thrombopoietin receptor agonists (TPO-RA) in selected conditions.

#### Platelet transfusions

Prophylactic platelet transfusions are recommended by oncology guidelines with a platelet threshold of <10 × 10^9^/L in all patients to reduce the bleeding risk, independently of antithrombotic therapy, based on phase III studies.^12,13,62^ In case of major bleeding or invasive procedures with a high-bleeding risk, higher platelet transfusion thresholds (generally ≥50 × 10^9^/L) are recommended.^62^

Although this is a relatively common practice, the efficacy and safety of increased platelet transfusion thresholds/targets, while on therapeutic anticoagulation is not proven.^63–66^ In a recent observational prospective study of cancer patients with acute VTE and TP, the most common platelet transfusion threshold among the 75 patients starting full-dose anticoagulation was <50 × 10^9^/L (74%),^67^ likely reflecting the rationale of using platelet transfusions to reach the minimal platelet count threshold allowing antithrombotic therapy, which is usually 40–50 × 10^9^/L.

Patients at very-high-thrombotic risk or with acute thrombosis may theoretically benefit from such a strategy for a short time. Such patients include those with grade 3–4 TP and mechanical heart valves, acute proximal lower extremity deep vein thrombosis (DVT), pulmonary embolism (PE), or ACS.^4^ On the other hand, the 50 × 10^9^/L threshold to enable antithrombotic drug treatment is associated with increased utilization of platelet units and depletion of stores,^64,65,68^ increased costs, refractoriness to future platelet transfusion, and potential adverse effects, including arterial and venous thrombosis.^65,68–70^ In addition, a high transfusion threshold was associated with early discontinuation of anticoagulation in as many as 36% of patients within 30 days of VTE because of difficulty achieving the transfusion goal of 50 × 10^9^/L platelets.^64,65,71^

**Recommendations**

**3. Use of platelet transfusion**

a. Grade 3–4 TP:
  **i.** Platelet transfusion with a target of 40–50 × 10^9^/L together with therapeutic-dose LMWH, if the platelet target is achieved, may be considered in patients with very-high-thrombotic risk* for a maximum of 14 days. We recommend against this as a routine approach. If the platelet target is not reached, but platelet counts of 25–40 × 10^9^/L are achieved, reduced-dose LMWH may be considered according to specific recommendations (5.b.ii and 5.b.iv). ***Level 5, grade D***
  **ii.** Platelet transfusion support to achieve platelets >25 × 10^9^/L may be considered to enable low-dose aspirin in extreme scenarios, such as a coronary lesion causing cardiogenic shock or in situations of very-high CV risk (**Table 3**). This should be only considered as a bridging strategy when the anticipated duration is limited to up to 7–14 days. If the desired platelet threshold is not achieved, APT should be held. ***Level 5, grade D***

*Mechanical heart valves, acute PE, acute lower extremity proximal DVT, ACS, or atrial fibrillation (AF) with arterial thromboembolism in the previous month.

#### TPO-RA

TPO-RA (eltrombopag, romiplostin, avatrombopag, and lusutrombopag) have been approved for immune and cirrhosis- or aplastic-anemia-associated TP. Thus, their use in the cancer-TP setting is currently off-label. Retrospective,^72^ phase 2 studies (including 1 randomized phase 2 study^73^) and 1 phase 3 trial^74^ have investigated TPO-RAs for the treatment and secondary prevention of chemotherapy-induced TP in solid tumors.^75^ The majority of evidence comes from studies of subcutaneous romiplostim given once weekly at doses titrated up to 10 µg/kg/wk according to platelet counts. In the largest retrospective study to date (n = 173 [153 solid tumor and 20 lymphoma or myeloma]), 71% of patients receiving romiplostim achieved a platelet response, and 89% avoided platelet transfusions.^72^ Predictors of nonresponse included bone marrow tumor invasion, prior pelvic irradiation, and exposure to temozolomide.^72^ A recent phase 3 placebo-controlled trial of avatrombopag for chemotherapy-induced TP in nonhematological malignancy did not achieve the primary efficacy endpoint, but avatrombopag appeared to be safe and was able to augment platelet counts.^74^

As summarized in a recent review, the rate of thrombotic complications in cancer patients who received romiplostim was between 5% and 15% in phase 2 and retrospective cohort studies (most without comparison groups), which is comparable to expected rates in cancer patients.^75,76^ Most of the events were VTE and only a small number of arterial events were reported. The placebo-controlled study of avatrombopag for chemotherapy-induced TP did not raise any thrombotic safety signals; thromboembolic events occurred in 2 (2%) patients receiving avatrombopag and in 1 (3%) patient receiving placebo.^74^ Of note, this study excluded patients with cardiovascular disease or arterial or venous thrombosis within 3 months of screening. In addition, among adults with immune thrombocytopenia, thrombotic complications appear to be slightly more frequent in those receiving TPO-RAs.^77^ Accordingly, concerns remain regarding the potential of TPO-RAs to increase thrombosis in patients with cancer. In one study of 302 immune thrombocytopenia patients receiving eltrombopag, 3 of the 19 cases of thrombosis occurred during periods of thrombocytosis, generating the hypothesis that thrombocytosis may be associated with a higher thrombotic risk.^78^ Therefore, we suggest adequate dosing of TPO-RAs to avoid thrombocytosis.

Both romiplostim and eltrombopag have been studied in patients with myelodysplastic syndrome (MDS), acute myeloid leukemia, and postallogeneic HSCT.^75^ Romiplostim showed a potential benefit in patients with grade 4 TP related to low-risk MDS^79^ and postallogeneic HSCT.^80^ Similar results have been shown with eltrombopag in patients with low-risk MDS or postallogeneic HSCT.^81–84^ TPO-RAs may carry a risk of progression to acute leukemia in patients with high-risk MDS in combination with azacitidine^85^ and a risk of serious adverse events and deaths caused by hemorrhage in patients with acute myeloid leukemia undergoing induction chemotherapy.^86^

**Recommendations**

**4. Use of TPO-RA**

a. Grade 3–4 TP:
  **i.** TPO-RA may be used in patients with anticipated long duration of TP, providing that the patient does not have high-thrombotic risk, acute leukemia, MDS, or extensive bone marrow infiltration. ***Level 5, grade C***

## ANTICOAGULANT THERAPY

### Therapeutic doses of parenteral and oral anticoagulation

This section considers patients with the following indications for oral or parenteral anticoagulant therapy: VTE; nonvalvular atrial fibrillation (nvAF), and mechanical heart valves. Major RCT on anticoagulation for cancer-associated thrombosis or nvAF excluded patients with TP between 50 and 100 × 10^9^/L.^33,34,59,87,88^ Therefore, evidence on anticoagulation and high degrees of TP stems from case series and cohort studies, largely retrospective and limited primarily to VTE and LMWH. In this section, “anticoagulation” refers to both oral and parenteral anticoagulation. When specific anticoagulation classes are referred to, this is specified.

#### Thrombosis versus bleeding risk conditions

**Table 5** details the studies, largely retrospective, of patients with cancer, grade 1–4 TP and anticoagulation for VTE, and shows variable bleeding and thrombotic rates. An important guide for management decisions is a high short-term risk of major or clinically relevant bleeding, especially with therapeutic-dose anticoagulation.^11,13,16,63,65,71,89^ Accordingly, the thrombotic risk should be sufficiently high to justify continuing therapeutic-dose anticoagulation. The first month post-VTE is a high-period risk for both recurrent thrombosis and bleeding^4,90^ (**Table 5**). Higher VTE burden (eg, PE or proximal lower extremity DVT) is also considered at higher risk for extension or recurrence.^91^ Cancer-associated incidental PE is associated with a high 12-month VTE recurrence rate (6% [95% CI, 4.4%-8.1%]) despite anticoagulation, and incidental subsegmental PE appears to have a VTE recurrence rate (6.4%) similar to more proximal incidental PE.^92^ Accordingly, incidental and subsegmental PE should not be classified as lower risk events in cancer patients.

**Table 5.** Cohort Studies of TP Cancer Patients Receiving Anticoagulation for VTE

Type of Cancer (N), Citation	PLT Cutoff at Index (× 10^9^/L)	Time since VTE, % of patients		UE-DVT[CR]	Index Event	FU for Outcome (d)	AC Management at Index			Bleeding			Recurrent/new VTE^a^	
		<1 mo	≥1 mo				% Continue^b^ (Full; Reduced)	% Hold	PLT (× 10^9^/L) Transfusion Target for “Full”	Continue^b^ (Full; Reduced)	Hold	Bleeding Definition	Continue^b^ (Full; Reduced)	Hold
Solid and hematological malignancies														
Any cancer (n = 74; 76% HM)^62^	<100 (median 50, IQR 28–78)	100%	0%	42% [38]	VTE	90	77% (41%; 36%)	23%	NS/mixed	19%^c^(7%; 33%)	12%	CSB	26% ^c^(10%; 44%)	47%
Any cancer (n = 99; 59% HM)^53^	<50 (≥7 d)	NS (median days to TP, 89.5)		26% [NS]	NS	NS	19% (5%;14%)	63%	NS	9.3%		CRNMB	0%	0%
							18% mix reduce/hold			0%		MB		
Any cancer (n = 121; 70% HM)^66^	<100	100%	0%	40% [31]	VTE	60	89% (62%; 27%)	11%	50 (in 74%)	9% (11%; 6%)	0%	MB	4% (5%; 0%)	8%
Any cancer (n = 877; 18% HM)^93^	50–99	100%	0%	NS	VTE	30	97% (87%; 10%)	3%	NS	4.4%		MB	2.1%	
	<50	100%	0%	NS	VTE	30	90% (57%; 33%)	10%	NS	2.4%		MB	2.4%	
Hematological malignancies only														
HM (n = 47)^16^	<50	100%	0%	36% [NS]	VTE	NS (median FU, 24.6 mo)	79% (32%; 47%)	19%	10	13.5% (26.7%; 4.5%)	0%	CSB	21.6% (33.3%; 13.6%)	22.2%
										6-mo CIR: 6.5% (95% CI, 2.2%-19.5%)			1 y CIR: 14.8% (95% CI, 7%-30.9%)	
HM (n = 78)^54^	<50^d^	59%	41%	74% [67]	TP	100	58% (55%; 3%)	42%	40–50	27%^c^	3%	CSB	2%^g^	15%
HM (n = 82)^36^	<50 (≥3 d)	100%	0%	77% [77]	VTE	30	79% (79%; 0%)	21%	50 (met in >85%)	48.3%	29.2%	WHO 2–4	10.8%	0%
										15.5%	0%	MB		
Acute leukemia, (n = 74)^67^	≤50	100%	0%	0% [0]	VTE	365	31%^f^(4%; 27%)	69%^g^	NS	17.4%^e^	9.8%	CRNMB	2.7%	
										21.7%^e^	29.4%	MB		
Stem cell transplantation														
Autologous HSCT (n = 204)^63^	None^d^^,h^	35%	65%(31% >3 mo)	55% [55]	HSCT	30	65%^i^(65%; 0%)	35%	50–70	41%^e^	32%	WHO 2–4	1.5%^g^	1.4%
										3.8%^e^	4.2%	MB		
Autologous and allogeneic HSCT (n = 340)^94^^j^	≤50	29%	71%(40% >3 mo)	49% [49]	TP	30	67%^i^(67%; 0%)	33%	NS	41%^e^	31%	WHO 1–4	3%^g^	4%
										7%^e^	5%	MB		

This table summarizes key data from analytical studies (all retrospective, expect for one prospective study^95^) of patients with cancer, TP (<100 × 10^9^/L) and AC prescribed for VTE. Only studies reporting bleeding and thrombotic outcomes according to management were included. One study^53^ had a potential selection bias toward patients with lower bleeding risk since at least 1 dose of full therapeutic-dose LMWH, before platelet nadir, was required for inclusion.

^a^VTE which was either progressive, recurrent or new, using varying definitions.

^b^Continue full *or* reduced-dose AC intensity, density or duration. In some studies, the “reduced” group received prophylactic dose LMWH^16^ and in others they received intermediate LMWH or unfractionated heparin doses,^53,67^ whereas sometimes the strategies were mixed^54,62,93^ or not specified. The vast majority of AC used was LMWH or unfractionated heparin.

^c^Statistically significant difference in outcomes compared to the hold AC group (*P* < 0.05).

^d^Outcomes were stratified/adjusted based on platelet levels over time. The other studies only used platelet count at diagnosis.

^e^*No* statistically significant difference in outcomes compared to the hold AC group (*P* ≥ 0.05).

^h^Some patients had AC held when platelets were below 25 × 10^9^/L.

^f^21 of 51 patients who had AC held (28% of all patients), had an IVCF inserted. AC was held in some cases when PLT < 25 × 10^9^/L in the “continue” group.

^g^All levels of platelet counts were included at index, but subsequent TP was universal, after conditioning for HSCT.

^h^Full AC was defined as AC continued for ≥3 d with adequate transfusion support.

^i^IPW was used for adjustment to balance confounders. All results, except management distribution, are shown after IPW.

AC = anticoagulation; CIR = cumulative incidence rate; CR = catheter related; CRNMB = clinically relevant nonmajor bleeding; CSB = clinically significant bleeding; FU = follow-up; HM = hematological malignancy; HSCT = hematopoietic stem cell transplantation; IPW = inverse-probability weighting; IQR = inter-quartile range; IVCF = inferior vena cava filter; LMWH = low-molecular weight heparin; MB = major bleeding; NS = not specified; PLT = platelets; TP = thrombocytopenia; UE-DVT = upper extremity deep vein thrombosis; VTE = venous thromboembolism.

The CHA_2_DS_2_-VASc (congestive heart failure, hypertension, age ≥75 y, diabetes mellitus, stroke or TIA, vascular disease, age 65 to 74 y, sex category) score may be used to assess the thrombotic risk in patients with nvAF.^59^

Lower thrombotic risk conditions include catheter-related upper extremity DVT (UE-DVT)^96^; VTE >90 days, subacute VTE (30–90 d since the event),^64^ and low-intermediate risk AF (no previous thromboembolism and CHA_2_DS_2_-VASc score < 4).^94^ Isolated distal lower-limb DVT is classified at lower risk by the ISTH,^91^ but recent retrospective data suggests VTE recurrence rates similar to proximal DVT in cancer patients.^97,98^

#### Adjustment of anticoagulation

When platelets are >50 × 10^9^/L (eg, grade 1 and 2 TP), therapeutic-dose anticoagulation, either oral or parenteral, appears safe^16^ since the bleeding risk increases for platelets <50 × 10^9^/L as compared to ≥50 × 10^9^/L.^16,36^ However, it is unclear whether thresholds below 50 × 10^9^/L may differently affect safety or efficacy.^17,63,64^ For instance, in a well-designed retrospective study, neither proposed platelet transfusion threshold nor platelet count (stratified into platelet count categories using the area under the curve from all platelet counts during follow-up) were predictive of bleeding in patients with anticoagulation and TP postautologous HSCT.^64^ Factors associated with bleeding in TP cancer patients are detailed in **Table 4**, including allogeneic or autologous HSCT,^52^ and renal or liver dysfunction.^64^

**Figure 1.**
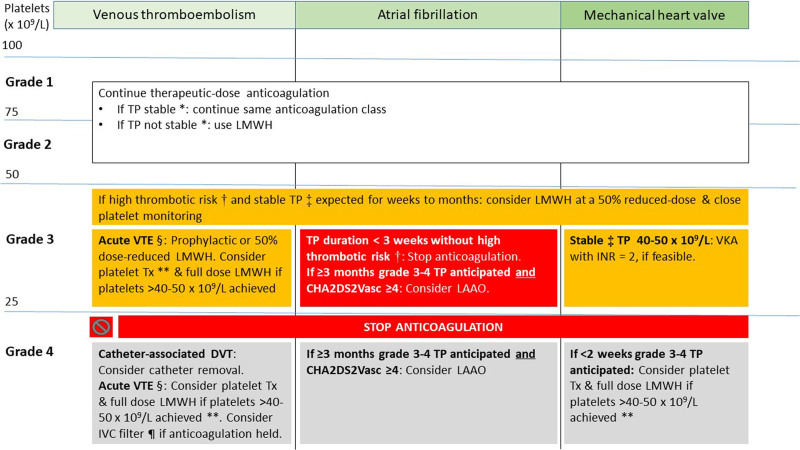
**Management of anticoagulation in cancer patients with TP.** *Stable grade 1–2 TP is defined as platelet counts, which are not expected to decrease to grade 3–4 TP in the coming days to weeks. †AF with arterial thromboembolism in the past 3 mo; AF with CHA2DS2-VASc ≥ 6; VTE in past 3 mo; mechanical heart valves where full-dose anticoagulation was not possible. ‡Stable grade 3 TP defined as platelet counts, which are not expected to decrease to grade 4 TP in the coming days to weeks. §VTE within the past 30 d. ¶Only in case of lower extremity DVT or pulmonary embolism. **This strategy can be used for a maximum of 14 d. AF = atrial fibrillation; DVT = deep vein thrombosis; INR = international normalized ratio; LAAO = left atrial appendage occlusion; LMWH = low-molecular weight heparin; TP = thrombocytopenia; Tx = transfusion; VKA = vitamin K antagonist; VTE = venous thromboembolism.

Accordingly, we recommend therapeutic-dose anticoagulation in most patients with grade 1–2 TP who have an indication for anticoagulation post-VTE (**Figure 1**). In patients with AF and grade 1–2 TP, we recommend continuing ongoing anticoagulation without changing dose, by extrapolation from safety data on VTE,^16^ since there is no specific evidence. The anticoagulation dose should be further assessed on a case-by-case basis if additional bleeding risk factors exist (**Table 4**). Full-dose anticoagulation should not be used in patients with grade 3–4 TP.

In specific patients with mechanical heart valve and stable platelet counts between 40 and 50 × 10^9^/L lasting weeks to months, we recommend considering VKA with close monitoring of both platelet counts and international normalized ratio (INR) target of 2.0. This recommendation considers the high risk of prosthetic thrombosis and systemic emboli (8.6%/y) associated with mechanical heart valves not receiving anticoagulation for extended periods of time.^99^ DOACs are always contraindicated for mechanical heart valves, independently of AF.

Several cohort studies have shown that VTE frequently recurs soon after platelet count recovers in patients who do not restart anticoagulation.^52,71^ A retrospective study of 250 patients postallogeneic HSCT demonstrated a relative 20% increase in VTE recurrence in patients with prior VTE (most subacute or remote) who did not restart anticoagulation after platelet engraftment, whereas the VTE rates were low during periods of grade 3–4 TP (median 14 d of grade 3–4 TP).^52^ Therefore, among patients who have anticoagulation held or reduced during TP, we recommend resuming full-dose anticoagulation as soon as platelet count allows, if the indication persists.

#### Anticoagulation class

When anticoagulant therapy is continued, we recommend using LMWH over VKA or DOACs for all indications, except for specific patients with stable grade 1–2 TP and nvAF (in whom DOACs are safer) or mechanical heart valves or valvular AF (in whom VKA are indicated). Grade 1–2 TP is considered stable when it is not expected to decrease to grade 3–4 TP (see: *Assessing the risk of TP in patients receiving antithrombotic therapy* section).

The rationale supporting LMWH over VKA or a DOAC in most cases of grade 1 and 2 TP, includes more evidence on LMWH in this setting, higher bleeding rates with DOACs compared to dalteparin in cancer-associated venous thrombosis,^87,88^ shorter half-life and more feasible dose reduction.^67^

It is worth noting that there is not enough evidence with LMWH for stroke prevention in AF with no prospective randomized or observational studies. Based on a recent meta-analysis in patients with AF and cancer, DOACs were associated with significantly lower rates of thromboembolic events and major bleeding versus VKA.^100^ In oncology, similar rates of bleeding were observed between VKA and rivaroxaban or dabigatran, whereas apixaban showed significantly lower rates of bleeding.^101^ Therefore, in case of stable grade 1–2 TP, full-dose DOAC should be preferred over VKA or LMWH in cancer patients with nvAF and a CHA_2_DS_2_-VASC score which warrants continued anticoagulation, according to current guidelines.^59,102^

#### Anticoagulation dose reduction

In acute VTE and grade 3 TP, a reduced dose of anticoagulant therapy could reduce the bleeding risk.

Reduced-dose anticoagulation for acute VTE includes prophylactic fixed dose LMWH (eg, enoxaparin 40 mg once daily), and intermediate dose LMWH, that is between therapeutic and prophylactic doses (eg, 1 mg/kg enoxaparin once daily, or 0.5 mg/kg enoxaparin twice daily).^67^

Thus, LMWH given at fixed subtherapeutic dose in substitution to VKA because of invasive procedures or chemotherapy-induced TP, appears feasible and safe.^103^ LMWH was reinitiated 12/24 h after obtaining a stable platelet count ≥30 × 10^9^/L.^103^ Furthermore, studies on reduced-dose LMWH (ie, prophylactic fixed dose or 5U/kg/h continuous infusion) for veno-occlusive disease suggest that the bleeding risk is low.^93,95^ The efficacy (ie, thrombotic prevention) of dose reduction for VTE and grade 3 TP remains unclear based on a systematic review of 2 retrospective studies.^104^

Three additional studies showed somewhat conflicting results. In a retrospective cohort of acute leukemia, 23 cases of catheter-related thrombosis (CRT) were managed with platelet count-adjusted LMWH dose reductions.^91,96^ Events were low in this study since the cohort was small, and CRT may carry a lower thrombotic risk as well. In 166 patients in the RIETE registry with active cancer, acute VTE and grade 3–4 TP, the 30-day rates of major bleeding were similar in those with reduced (<100 IU/kg/d) and therapeutic (≥100 IU/kg/d) LMWH doses (3.4% and 2.9%, *P* = 0.86). In contrast, VTE recurrence rate was nonsignificantly higher in patients on reduced as compared to therapeutic LMWH doses (10.3% [3/29] versus 1.4% [1/70], respectively, *P* = 0.08); however, the number of events (3 versus 1) is too low to draw any sound conclusion.^105^ Finally, a recent prospective observational study of 121 patients with active cancer, acute VTE and any TP grade demonstrated a higher 60-day incidence of major bleeding among the 75 patients initially receiving full-dose LMWH than in the 33 who received reduced-dose anticoagulation (12.8% versus 6.6%; respectively; hazard ratio (HR), 2.18; 95% CI, 1.21-3.93).^67^ The cumulative incidence of recurrent VTE at 60 days was 5.6% in the full-dose group and none in the modified-dose group. This suggests that a reduced-dose anticoagulation could be safe and possibly effective in patients with cancer who develop DVT and TP. The median platelet count was 65 (IQR 47–88) × 10^9^/L in the full-dose group and 37 (24–48) in patients receiving modified doses. Of note, the index VTE in the modified-dose group (as compared to the full-dose group) was more frequently UE-DVT (51% versus 31%) and less frequently PE (21% versus 48%). Thus, considering the conflicting results of the above studies, there is still a substantial lack of evidence in terms of benefit/risk balance for these patients.

A recent retrospective cohort study of 61 hospitalized patients with hematological malignancy, AF and platelets <50 × 10^9^/L, 69% with no anticoagulation, demonstrated high 30-day bleeding (major and clinically relevant non major) incidence (13%; 95% CI, 6%-26%) and low incidence of arterial thromboembolism (3%; 95% CI, 0.4%-12%).^94^ This suggests that the bleeding may outweigh the thrombotic risk in AF patients with grade 3–4 TP, but more and better-quality evidence is needed.

**Recommendations**

**5. Management of therapeutic dose of oral and parenteral anticoagulation**

a. Grade 1–2 TP:
  **i.** We recommend therapeutic-dose parenteral or oral anticoagulation according to the approved indications after a careful evaluation of bleeding and thrombotic risk in the individual patient. ***Level 2b, grade C***
  **ii.** In patients with grade 1–2 TP, which is not stable* and acute VTE, LMWH should be preferred over DOACs and VKAs. ***Level 5, grade D***
  **iii.** In patients with grade 1–2 TP, which is not stable* and AF or mechanical heart valves, LMWH may be a temporary short-term option. ***Level 5, grade D***
b. Grade 3 TP:
  **i.** We recommend against using DOACs and VKAs for VTE and AF. ***Level 5, grade D***
  **ii.** LMWH, at doses either prophylactic or therapeutic reduced by 50%, should be used in patients with acute VTE, after balancing bleeding and thrombosis risk. ***Level 2b, grade C***
  **iii.** We recommend for holding ongoing anticoagulation in patients with AF if a short TP duration is expected, unless the patient is at very-high-thrombotic risk** or with additional cancer-related risk factors. ***Level 4, grade C***
  **iv.** Therapeutic LMWH dose reduced by 50% with close monitoring of platelet counts may be considered in patients, with stable grade 3 TP lasting weeks to months, at very-high-thrombotic risk**. ***Level 5, grade D***
  v. In patients with stable platelet counts of 40–50 × 10^9^/L and a mechanical heart valve, VKA should be considered, with a target INR of 2. ***Level 5, grade C***
  vi. In patients with mechanical heart valve and platelet count 25–40 × 10^9^/L or unstable INR, therapeutic LMWH dose reduced by 50% may be considered. ***Level 5, grade C***
c. Grade 4 TP: We recommend holding anticoagulant drugs for all the indications. ***Level 2b, grade C***
d. Grade 3–4 TP: In case of very-high-thrombotic risk, we suggest continuing anticoagulation and increase platelet counts by platelet transfusion or use of TPO-RA (see recommendation 3.a and 4.a). ***Level 5, grade D***
e. We recommend resuming the appropriate dose of anticoagulation as soon as platelet count allows. ***Level 2b, grade B***

* Stable grade 1–2 TP is defined as platelet counts, which are not expected to decrease to grade 3–4 TP in the coming days to weeks (see C.2).

** AF with arterial thromboembolism in the past 3 months; AF with CHA2DS2-VASc ≥6; mechanical heart valves where full-dose anticoagulation was not possible.

#### Holding Anticoagulant Therapy and Use of Medical Devices or Invasive Strategies

##### Inferior vena cava filters in tp cancer patients

There are no clinical trials on inferior vena cava filters (IVCF) placement in cancer patients with a contraindication to anticoagulation. A population-based study of cancer patients with acute lower extremity DVT demonstrated an improvement in PE-free survival on long-term follow-up in patients with IVCF insertion (for any indication) compared to those without (HR, 0.69; 95% CI, 0.64-0.75).^106^ The proportion of new DVT among patients who received IVCF was slightly lower than in patients who did not receive IVCF (18.7% versus 22.1%; respectively, *P* < 0.001). A prospective cohort study of cancer patients with acute PE or lower extremity DVT used propensity score matching to compare 30-day outcomes between patients with IVCF placement because of a significant bleeding risk versus patients without IVCF placement.^107^ PE-related mortality was lower with than without filter insertion (0.8% versus 4.0%; respectively, absolute reduction –3.2% [95% CI, –6.5% to –0.5%]). The recurrent VTE rate was higher in the IVCF group than in patients without IVCF (7.3% versus 3.2%; absolute increase 4.1% [95% CI, 0–8.3%]. Major bleeding did not differ significantly between the 2 groups (6.1% versus 5.7%; risk difference 0.4% [95% CI, –3.9% to 4.7%]). Although 14.2% of patients receiving IVCF had grade 1–4 TP (compared to 15.4% in those without IVCF, after matching), there is no data on outcomes in the subgroup of patients with TP in this study or other studies.^107^ Accordingly, also the procedure-related risk of bleeding associated with IVCF insertion in this specific population is not known.

Taken together, these studies suggest that IVCF placement may results in improved PE-free survival and PE-related mortality in cancer patients with lower extremity DVT or acute PE,^106,107^ with the potential risk of increased VTE recurrence^107^ and no data on procedure-related bleeding. There is still a substantial lack of evidence, especially in cancer patients with TP.

##### Intermittent pneumatic compression

In the general population, intermittent pneumatic compression devices or graduated compression stockings may be considered in patients with an indication for primary or secondary VTE prophylaxis in the absence of acute DVT, until pharmacological prophylaxis can be initiated, based on a grade 2C recommendations from the American College of Chest Physicians guidelines.^108^

##### Central venous catheter removal

Since a central venous catheter (CVC) is a major driver of CRT, catheter removal is often considered in patients with CRT who cannot receive anticoagulation. A multicenter retrospective cohort study assessed management of CVC and anticoagulation in 663 patients with hematologic malignancies and CRT.^109^ Catheters were removed because of UE-DVT in 392 (68%) patients,^109^ despite the current guideline recommending catheter removal only in case of catheter malfunction, completion of therapy, or infection.^110^ Median platelet count at baseline was 103.5 × 10^9^/L (IQR 44–193) overall, but lower in patients who did not receive anticoagulation, with (31 × 10^9^/L [18–83]) or without (30.5 × 10^9^/L [16–72]) catheter removal.^109^ There was an overall 15% rate of recurrent VTE after a median of 60 (IQR 15–167.5) days since the initial CRT. After adjustment for several potential confounders (including baseline platelet count), the 119 (18%) patients treated with catheter removal only (without anticoagulation) had an increased risk of VTE recurrence (HR 2.50 [95% CI, 1.24–5.07]) compared with patients continuing anticoagulation. This suggests that catheter removal alone does not suffice. Treatment group was not associated with VTE recurrence after adjusting for the competing risk of death. The 32 (5%) of patients who had neither anticoagulation nor catheter removal, had a high rate of death and nonsignificant increase in VTE.^109^ Therefore, we recommend resuming anticoagulation in patients with CRT who had their catheter removed before completing 3 months of anticoagulation, when platelet count and bleeding risk allow.

A small single center study (n = 83) reported similar findings in 62 (75%) patients with catheter removal alone, whereby 6.4% developed a recurrent VTE and 8% developed progressive symptoms leading to initiation of anticoagulation.^111^ Platelet counts dropped below 50 × 10^9^/L in 50% of patients with catheter removal alone compared to 14.2% of patients with catheter removal and anticoagulation. Bleeding rates were significantly higher in patients who received anticoagulation (28.5% versus 4.8%; *P* = 0.007).

Taken together, the above studies show that catheter removal is common practice in cancer patients with CRT and that catheter removal without anticoagulation may achieve a reasonable balance between recurrent thrombosis and bleeding for grade 4 TP. We recommend assessing the clinical utility of the CVC in patients with CRT who cannot receive anticoagulation. We recommend considering removal of CVC in patients with acute UE-DVT and CRT (within 30 d) who cannot receive anticoagulation, depending on the indication for the CVC and the importance of central venous access. Longer anticipated durations of grade 3–4 TP (eg, >7–14 d) and a greater DVT symptom load, strengthen this statement.

Whether CVCs can be removed early (ie, within 48 h from starting anticoagulation) is debated. A previous consensus-based following an acute ischemic on CRT suggested at least 3 to 5 days of anticoagulation before CVC removal for UE-DVT, not evidence-based.^110^ However, a recent study of 626 patients with acute CRT showed that early (≤48 h) removal of CVCs (with and without anticoagulation) was not associated with an increased risk of PE within 7 days compared with delayed (>48 h) or no removal (0.78% versus 0.44%; respectively, *P* > 0.9).^112^ All 3 patients with PE had brachiocephalic vein or superior vena cava thrombosis as the index CRT. Therefore, in patients with brachiocephalic vein or superior vena cava thrombosis, the benefit of early removal should be carefully weighed against a potential thromboembolic risk.

##### Left atrial appendage occlusion in AF

According to the EHRA/EAPCI expert consensus statement on catheter-based left atrial appendage occlusion (LAAO), there are possible clinical scenarios in which LAAO may be a reasonable option for the prevention of stroke and embolism in patients with TP^113^; however, this document did not include TP patients with AF, consistently excluded from clinical registries.

In a general AF population at high risk of bleeding (not specific because of TP and cancer), the PROTECT AF & PREVAIL studies demonstrated that LAAO is as effective as VKA for preventing stroke and death because of cardiovascular or unexplained causes in AF.^114–116^ It should be highlighted that warfarin was administered for 45 days immediately after the LAAO, followed by dual APT (DAPT) and then aspirin as life-long single APT (SAPT). It is important to consider the necessity and feasibility for post-interventional antithrombotic therapy when considering LAAO for cancer patients with TP.

The most effective and safe post-interventional antithrombotic prophylaxis and its duration after LAAO is not defined and has never been evaluated by RCTs. In patients with a contraindication for oral anticoagulants (OAC) because of elevated risk of bleeding, DAPT (aspirin plus clopidogrel) is commonly prescribed for at least 1 month and up to 6 months, with antiplatelet therapy modified if necessary (eg, downgrading to SAPT).^117,118^ Complete LAAO and the absence of device surface thrombi may enable early switching to SAPT.

Although not specific to TP, additional evidence on the efficacy and safety of LAAO can be found in the following studies: ASAP including only patients with a clear contraindication to warfarin^119^; EWOLUTION registry of patients with moderate-to-high risk of bleeding (average HAS-BLED score: 2.3 ± 1.2; 72% with a clear VKA contraindication, who underwent SAPT, DAPT, VKA, or no antithrombotic-only 6%-after the procedure)^120^; cohorts of patients after intracerebral hemorrhage^121^ or major GI bleeding^122^ or with severe kidney disease.^123^

There is only 1 retrospective study with long-term follow-up investigating the feasibility, efficacy, and safety of LAAO in TP patients.^124^ Propensity scores were used to match 32 patients with platelets <10 × 10^9^/L with 160 control subjects. LAAO was safely performed; none of the TP patients experienced ischemic stroke or systemic embolization, whereas 1 (3%) had device-related thrombus. There was a higher risk of major bleeding in the TP group (12.5% versus 3.75%; *P* = 0.06), but it is worth noting that 4/4 major and 4/5 minor bleeding events in the TP group occurred during anticoagulation or DAPT after procedure. There was only 1 minor gingival bleeding during 1-year of aspirin SAPT. An important limitation of this study was the absence of details on platelet counts in the control and TP group (at index, during follow-up, and at the time of events).

Taken together, a LAAO would be justified only in case of long-term severe TP (eg, grade 3–4) and very-high-thrombotic risk (**Table 3**). Accordingly, we recommend carefully considering LAAO in patients with grade 3–4 TP with an anticipated duration of months to years if the CHA_2_DS_2_-VASc score is ≥4 and if aspirin can be administered for a minimum period of 2–4 wks after LAAO. A minimum expected 3-month duration of grade 3–4 TP is proposed. The rationale behind this is that the first several months would usually necessitate DAPT, meaning that the benefit (with respect to bleeding risk) of no or low-intensity antithrombotic therapy (ie, SAPT alone) would be emphasized especially after the first 3–6 months. The indication for LAAO would be strengthened by the presence of additional bleeding risk factors (**Table 4**), additional thrombotic risk factors (**Table 3**), including higher CHA_2_DS_2_-VASc scores or prior stroke, and expected reasonable life expectancy (eg, >12 mo). An example of a LAAO candidate, is a 65-year-old patient with a CHA_2_DS_2_-VASc score of 5 (including prior embolic stroke) and low-risk MDS and grade 3 TP. For patients who may not tolerate any (S)APT, either an epicardial catheter approach or thoracoscopic clipping of the left atrial appendage might be options, whereas an endocardial device should not be implanted. If it is anticipated that SAPT cannot be administered for at least 2–4 wks after LAAO, epicardial closure (using either surgical intervention or the LARIAT device), should be considered in an experienced center.

**Recommendations**

**6. Device use when therapeutic-dose anticoagulation is held**

a. **IVCF** in patients with PE or lower extremity DVT
  **i.** Removable IVCF may be considered on an individual basis in patients with acute PE or acute lower extremity DVT, up to 30 days thereafter. ***Level 2b, grade B***
  **ii.** We recommend periodically reassessing the removal of IVCF and removing it whenever possible. ***Level 2b, grade C***
  **iii.** The contraindication for anticoagulation should be frequently (eg, weekly) reassessed in patients with IVCF. Anticoagulation should be restarted when the severe bleeding risk and TP resolve. ***Level 5, grade D***
  **iv.** IVCF are not recommended starting 30 days after VTE. ***Level 5, grade D***
  **v.** IVCF are not recommended for primary VTE prophylaxis. ***Level 5, grade D***
**b. Elastic or pneumatic compression**
  **i.** Intermittent pneumatic compression devices or graduated compression stockings may be considered in patients with an indication for secondary VTE prophylaxis in theabsence of acute DVT, until anticoagulation can be initiated. ***Level 5, grade D***
c. **CVC removal** in catheter-related UE-DVT
  **i.** Removal of the CVC is recommended in patients who cannot receive anticoagulation, within the first 30 days of an acute UEDVT. ***Level 4, grade C***
  **ii.** If the CVC is essential for patient care, the feasibility of placing a CVC in the other upper extremity should be assessed. ***Level 5, grade D***
  **iii.** For patients who have CVC removed after <3 months of anticoagulation, full-dose anticoagulation is recommended as soon as platelet count reaches ≥50 × 10^9^/L. ***Level 5, grade D***
d. **LAAO** in patients with AF
  i. LAAO may be considered in patients with AF and TP fulfilling all of the following:
   • Long-term grade 3–4 TP (at least 3 mo)
   • CHA_2_DS_2_-VASc score ≥4
   • No contraindication to low-dose aspirin for at least 2–4 wks after LAAO. ***Level 4, grade C***

ii. Antiplatelet therapy after LAAO:
** 1. **Grade 1–2 TP: DAPT for 6 months, then long-term aspirin. ***Level 5, grade D***
 2. Grade 3 TP: Long-term low-dose aspirin. If platelets ~40 to 50 × 10^9^/L, consider DAPT for at least 1 month. ***Level 5, grade D***
 3. Grade 4 TP: 2–4 wks of aspirin for platelet 20–25 × 10^9^/L. Otherwise, no APT. ***Level 5, grade D***

### Prophylactic Dose Anticoagulation

Primary thromboprophylaxis with LMWH is indicated in the majority of medical and surgical inpatients with cancer. Furthermore, a growing proportion of cancer outpatients are candidates for primary prophylaxis with LMWH or specific DOACs (apixaban or rivaroxaban).^125–127^ RCTs comparing LMWH with placebo for VTE prophylaxis in cancer outpatients excluded patients with platelet counts below 50 × 10^9^/L^128^ or 100 × 10^9^/L^129^ or LMWH was discontinued at platelet counts below 50 × 10^9^/L.^129^ The landmark trials on thromboprophylaxis with factor Xa oral anticoagulants, apixaban and rivaroxaban, in cancer outpatients with an intermediate-high VTE risk, excluded patients with platelet counts below 50 × 10^9^/L.^76,130^ Nonetheless, the risk of major bleeding was approximately doubled in patients receiving DOACs than in the placebo group. Prophylactic dose rivaroxaban was held per-protocol when platelet counts were below 25 × 10^9^/L for at least 1-wk, whereas management of apixaban with low platelet counts was not specified. There are no data on outcomes of patients with baseline or incident TP in these studies.

### Perioperative anticoagulation

Regarding elective surgery in cancer patients with an indication for OAC (eg, AF), it is likely that platelet counts warranted for surgical procedures (ie, at least 20–50 × 10^9^/L) would suffice for postoperative prophylactic dose LMWH.^125^ Concerning the pre-operative bridging from an OAC to LMWH, even though data on cancer-TP patients are lacking, the BRIDGE trial tested the noninferiority of interrupting VKA before surgery with LMWH-bridging versus without, in patients with AF, normal platelet counts, and a clear indication for VKA (mean CHADS_2_ score 2.3, ≥3 in 38%). Thromboembolism (both arterial and venous) at 30 days did not differ between the 2 arms, whereas ISTH-defined major bleeding significantly increased in the LMWH-bridging arm (RR 0.41; 95% CI, 0.2-0.7).^131^ Similar results were reported in an observational substudy of the RE-LY trial, which included warfarin and dabigatran with or without pre-surgery bridging.^132^ Although patients with platelets <100 × 10^9^/L were excluded from the BRIDGE study, trial data from non-TP subjects indicates that OAC (VKA and DAPT) can be safely interrupted perioperatively for a few days and resumed soon after surgery without bridging to further reduce the bleeding risk. Patients should be managed on an individual basis, balancing the bleeding risk of the type of surgery/procedure (low versus high)^131^ vis-à-vis additional patient-related bleeding risk factors (**Table 4**), likely independent of the degree of thrombosis risk in patients with a clear indication for OAC.

**Recommendations**

**7. Prophylaxis of venous thromboembolism**

**a.** Grade 1–2 TP: Standard prophylactic dose LMWH or standard prophylactic dose of apixaban or rivaroxaban should be used according to the current indications. ***Level 4, grade C***
**b.** Grade 3 TP: Standard prophylactic dose LMWH, not DOACs may be considered in the absence of additional bleeding risk factors (**Table 4**) and if platelet counts are stable* or can be monitored closely. ***Level 5, grade D***
**c.** Grade 4 TP: We recommend against any pharmacological VTE prophylaxis. ***Level 5, grade D***

*Stable grade 3 TP is defined as platelet counts which are not expected to decrease to grade 4 TP in the coming days to weeks.

## ANTIPLATELET THERAPY

### Single antiplatelet therapy

#### Secondary prevention

##### Aspirin

Low-dose aspirin, an irreversible inhibitor of platelet-derived thromboxane A_2_,^133^ as SAPT is the reference treatment to prevent recurrence of major CV events and CV death in patients with a previous MI, revascularization, stroke, transient ischemic attack (TIA), or symptomatic peripheral arterial disease (PAD).^134–136^ Since these patients are considered overall at very-high CV risk, based on a risk of fatal CV disease >1%/y,^137,138^ the degree of TP and other ongoing risk factors for bleeding should be weighed against their very-high risk of CV event recurrence and CV death (**Tables 3** and **4**). Importantly, no clinically relevant DDIs with chemotherapeutical drugs are known with aspirin, that is not biotransformed by the cytochrome p450 system.

In the very-high CV risk patients on secondary prevention, aspirin reduces the absolute rate of MI, stroke or vascular death by 1.5%/y (from 8.19 to 6.69%/y), whereas it increases the absolute rate of nonfatal extracranial bleeding by 0.19%/y (from 0.06 to 0.25%/y) mostly of GI origin (see: *General management of patients with antithrombotic therapy and thrombocytopenia* section), with a favorable benefit/risk profile (number needed to treat [NNT]: 66; number needed to harm [NNH]: 526).^10^

Considering that the hemorrhagic risk is relatively small for platelet counts >75 × 10^9^/L (grade 1 TP), even while on APT (**Table 6**), we advise to maintain low-dose aspirin for TP grade 1. For stable grade 2 TP (50–75 × 10^9^/L), we advise to continue low-dose aspirin in the absence of other ongoing major bleeding risk factors. This is supported by small observational studies showing no major bleeding complications for platelet counts >50 × 10^9^/L (**Table 6**).^20^ For patients with grade 3 TP (25–50 × 10^9^/L), we advise to withhold aspirin unless multiple CV risk factors are present or comorbidities predisposing to CV events (**Table 3**), in the absence of other bleeding risk factors (**Table 4**).^139^ In this setting also, the type of cancer and chemotherapy predisposing to a higher thrombotic risk should be considered (**Table 3**). A small study on patients with hematological cancers, known to have a higher thrombotic risk, and platelet count <50 × 10^9^/L,^20^ reported a significant benefit from low-dose aspirin on CV mortality at 3 years after an acute event (20% versus 60% of survival without and with aspirin, respectively), but this evidence is too limited to advise for always maintaining aspirin in grade 3 TP.

**Table 6.** Outcome Studies on Patients With Baseline TP and Acute Major Arterial Vascular Events

References	Study Design and Population	TP Degree and Origin	Ongoing APT	Bleeding Incidence	Thrombosis Incidence	Mortality
Sarkiss et al^169^	Observational, retrospective; ACS cancer patients with or without TP	TP: <100 × 10^9^/L (median 32 × 10^9^/L), n = 47 Non-TP: >100 × 10^9^/L, n = 43	ASA: 37% TP patients, 74% of non-TP patients	No differences in major bleeding in patients off vs on ASA. More bleeding on TP patients, but ASA-unrelated	NA	Overall 7-d death higher in no ASA vs ASA: OR 18.44 [2.87–118.60] Lack of beta-blocker use also associated with higher mortality
Overgaard et al^145^	Observational, retrospective; elective and urgent PCI	TP: <150 × 10^9^/L, n = 639, various origin; Non-TP: ≥150 × 10^9^/L, n = 10,182	Same DAPT/SAPT in TP and non-TP patients	Major bleeding: 1.7% TP vs 0.8% non-TP, *P* < 0.01 GI bleeding: 1.1% TP vs 0.5% non-TP, *P* < 0.05	NA	In-hospital mortality: 1.9% TP vs 0.6% non-TP, *P* < 0.01, mostly for urgent PCI OR 2.07; 95% CI, 1.1-4.1
Hakim et al^19^	Subgroups of RCT; STEMI patients undergoing PCI with and w/out TP at baseline	TP: <150 × 10^9^/L, n = 146; various origin Non-TP ≥ 150 × 10^9^/L, n = 3330	ASA: 98% at discharge in TP and non-TP Clopidogrel: 85.4% TP vs 92% non-TP, *P* < 0.05	Major bleeding: TP 15.4%, non-TP 9.1%, *P* = 0.01	2-y MACE: TP 24.7%, non-TP 18.5%, *P* = 0.03	2 y all-cause mortality: TP: 11.3%, non-TP 5.1%, *P* < 0.01; ASA at discharge: HR for 2-y mortality: 0.23 [0.09-0.59]
Sico et al^159^	Retrospective, Cohort Stroke patients	TP: <100 × 10^9^/L, n = 28; Various origin Non-TP: >100 × 10^9^/L, n = 1205	NA	Symptomatic poststroke intracranial bleeding: TP vs no TP: OR 17.6 [4.6-67]	NA	In-hospital mortality TP vs non-TP: OR 6.6 [2.3-18.6]
Yadav et al^146^	Subgroup of 2 randomized trials; NSTEMI and STEMI patients	TP: 100–150 × 10^9^/L, n = 607, various origin Non-TP: >150 × 10^9^/L, n = 9996	Aspirin 98% of both groups; Thienopyridine: 77% non-TP vs 73% TP, *P* = 0.03	NA	TP independent predictor of MACE: HR 1.39 [1.09-1.7] and ischemic TLR HR: 1.37 [1.04-1.81]	TP independent predictor of 1-y all-cause mortality: HR 1.74 [1.12-2.69]
Feher et al^20^	Observational, retrospective, MI patients with hematologic cancers	Mild TP: >50 × 10^9^/L, n = 58 Severe TP: ≤50 × 10^9^/L, n = 61 Cancer-related TP	ASA: 43% severe TP, 83% mild TP Thienopyridine: 3% severe TP, 27% mild TP	No difference in BARC 3–5 bleeding in the 2 groups	NA	Severe TP on ASA had improved survival at 7 d, 1 and 3 y vs severe TP w/out ASA, *P* = 0.008
Iliescu et al^163^	Observational, retrospective, ACS patients with cancer	TP: <100 × 10^9^/L, n = 98 as follows: 50–100 × 10^9^/L, n = 36; 30–50 × 10^9^/L, n = 20; <30 × 10^9^/L, n = 42 Cancer-related TP	No APT: 30.6% SAPT: 41.8% DAPT: 27.6% ASA (SAPT or DAPT): 67.3% Clopidogrel (SAPT or DAPT) 29.6%	No major bleeding over 72 mo	NA	Overall and CV mortality higher at lower platelet counts. Statins, ASA alone and DAPT associated with a trend of longer survival (*P* = 0.06)
Shiraishi et al^206^	Observational, retrospective; elective PCI	TP: 50–149 × 10^9^/L, n = 226 as follows TP mild: 100–149 × 10^9^/L, n = 187 TP moderate: 50–99 × 10^9^/L, n = 39; various origin non-TP: 150–449 × 10^9^/L, n = 1009	ASA: 87% in both TP and non-TP (moderate TP: 82%) Thienopyridines: 80% non-TP vs 73% TP (*P* < 0.05) (moderate TP 84.6%) DAPT: non-TP 75%, TP: 68.9% (*P* = 0.058)	No other differences in bleeding Access bleeding higher in moderate TP vs non-TP, OR 6.5 [1.7–24.2]	No differences in MACE	No differences in mortality among TP and non-TP and among TP mild and moderate
Long et al^24^	Meta-analysis, post-PCI (STEMI and NSTEMI) with TP at baseline	TP <100 × 10^9^/L, n = 37,753 Non-TP n = 81,192, various origins	DAPT	Postprocedural bleeding, GI bleeding, intracranial bleeding, major bleeding significantly higher in the TP group	MACE significantly higher in the TP group, whereas stent thrombosis was similar	NA
Liu et al^207^	Registry, STEMI patients, TP at baseline	TP <100 × 10^9^/L n = 364, various origin, as follows: 50–100 × 10^9^/L, n = 313; <50 × 10^9^/L, n = 51 non-TP, n = 16,593	ASA approximately 92% in TP and non-TP P2Y_12_ receptor antagonist approximately 98% in TP and non-TP *P* = ns	NA	Multivariable analysis showed no association between TP and MACE (HR 1.21 [0.89–1.63])	Multivariable analysis showed no association between 2-y all-cause death and TP, HR 1.21 [0.88-1.67]

ACS = acute coronary syndromes; ASA = aspirin; CV = cardiovascular; DAPT = dual antiplatelet therapy; GI = gastrointestinal; HR = hazard ratio; MACE = major adverse cardiovascular events; MI = myocardial infarction; NA = not available; ns = nonsignificant; OR = odds ratio; PCI = percutaneous coronary intervention; STEMI = ST elevation myocardial infarction; TP = thrombocytopenia.

**Figure 2.**
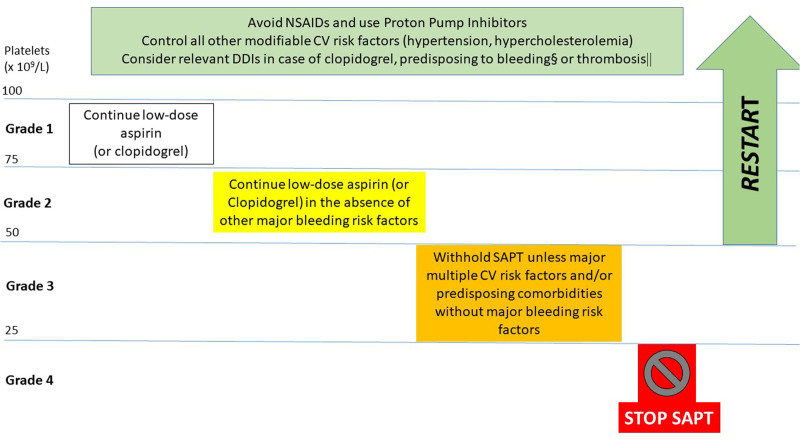
**Management of SAPT for secondary prevention in cancer patients with TP.** §Refer to **Table 4** for a nonexhaustive list of risk factors for major bleeding. ||Refer to **Table 3** for a nonexhaustive list of patients with high-thrombotic risk. CV = cardiovascular; DDI = drug-drug interaction; NSAID = nonsteroidal anti-inflammatory drugs; SAPT = single antiplatelet therapy; TP = thrombocytopenia.

Since the bleeding risk exponentially increases for grade 4 TP (<25 × 10^9^/L), likely exceeding any thrombotic risk, we suggest to temporarily withhold aspirin independently of all other risk factors, chemotherapy, or cancer type in stable patients with the following previous MI, coronary artery disease (CAD), revascularization >3 or 6 months because of chronic or ACS, respectively, stroke, TIA, or symptomatic PAD. There are sporadic reports on low-dose aspirin safely used in secondary prevention with platelet counts down to 10 × 10^9^/L, but numbers are extremely low.^20^ We advise to resume low-dose aspirin as soon as the platelet count raises to ≥50 × 10^9^/L and possibly ≥25 × 10^9^/L in patients with very-high-thrombotic risk. **Figure 2** summarizes the management of SAPT for secondary prevention in cancer patients with TP.

##### Clopidogrel

Clopidogrel, an irreversible inhibitor of the platelet P2Y_12_ receptor,^133^ is currently recommended as SAPT only in aspirin-intolerant patients with a previous MI, revascularization, stroke, TIA,^135,136>^ or as alternative to aspirin in symptomatic PAD.^134^ In a head-to-head comparison against aspirin in a large secondary prevention CV RCT,^147^ clopidogrel showed comparable major bleeding rate. A large registry study confirmed that GI bleeding complications are comparable between clopidogrel and aspirin.^148^ Thus, for clopidogrel, we advise the same decision-making strategy based upon the degree of TP, as depicted above for low-dose aspirin. However, clopidogrel is a prodrug with a complex cytocrome p450-dependent (3A4, 2B6, 2C19, 2C9) bioactivation, known to cause clinically relevant DDIs (Suppl. Table S1). DDIs with clopidogrel may either increase variability in its antiplatelet effect,^149^ or even increase the toxicity of chemotherapeutic agents such as taxanes.^150^ Consistently, we advise, if possible, to cautiously use clopidogrel in cancer patients on chemotherapeutical drugs, considering potential clinically relevant DDIs (Suppl. Table S1).

Beyond secondary prevention, low-dose aspirin is also the reference treatment in patients who have undergone revascularization for a significant arterial stenosis in the absence of a symptomatic MI or stroke and in patients with documented, clinically significant arterial stenosis (usually ≥50%).^135^ For these patients, deemed at very-high CV risk because of unequivocally documented relevant atherosclerotic disease, we advise to use the same reasoning pattern as in secondary prevention.

**Recommendations**

**8. SAPT in secondary prevention**

a. For grade 1 TP, we recommend to maintain SAPT with low-dose aspirin. ***Level 2b, grade B***
**b.** For grade 2 TP, we recommend to maintain SAPT with low-dose aspirin, providing that the patient has no other major bleeding risk factors (**Table 4**). ***Level 2b, grade C***
**c.** For grade 3 TP, SAPT with low-dose aspirin should be continued only if additional CV thrombotic risk factors are present (**Table 3**). ***Level 4, grade C***
**d.** Grade 4 TP, we recommend to temporarily withhold SAPT, independently of the thrombotic risk level. ***Level 5, grade D***
**e.** If withheld, SAPT should be resumed as soon as platelet count reaches ≥25 to 50 × 10^9^/L, according to the thrombotic risk. ***Level 5, grade D***

#### Primary prevention

In primary CV prevention low-dose aspirin use should be restricted to patients with multiple CV risk factors, estimated to be at high or very-high CV risk^137,151^or for patients with risk-enhancing factors (high Coronary Artery Calcium score) or in patients with no history of MI or stroke but with significant carotid and/or femoral plaque stenosis documented by imaging especially with diabetes.^151^ Considering that the absolute yearly risk of CV complications is lower in primary than secondary prevention, we advise to keep ongoing aspirin for grade 1 TP patients in the absence of other bleeding risk factors, and to temporarily withhold aspirin for grades 2–4, restarting the drug when platelets raise to >75 × 10^9^/L.

**Recommendations**

**9. Low-dose aspirin in primary prevention**

a. Grade 1 TP: we recommend maintaining SAPT with low-dose aspirin, if indicated, unless other bleeding risk factors are present. ***Level 5, grade D***
b. Grade ≥2: we recommend holding SAPT and resuming as soon as platelet count is ≥75 × 10^9^/L. ***Level 5, grade D***

### Dual antiplatelet therapy

Patients can be on DAPT (low-dose aspirin plus a P2Y_12_ inhibitor, either clopidogrel or prasugrel or ticagrelor) according to current guidelines^136,139^ for the following indications: acute minor ischemic stroke or high-risk TIA within the first 21 days; prior TIA/stroke with intracranial arterial culprit lesion within 90 days; percutaneous coronary intervention (PCI) and stent implantation for stable CAD within 6 months; ACS with or without revascularization within 12 months. Overall, there is scarce and low-quality evidence on patients with a clear indication for DAPT who have cancer and concurrent TP, especially grade 3–4. In addition to the bleeding risk in this setting (**Table 4**), the potential utility of DAPT in improving CV and cerebrovascular outcomes has been proven in RCT, which excluded TP patients. There is no evidence supporting the use of platelet function assays for any of the therapeutic decisions.^152,153^

#### DAPT in stroke prevention

Current stroke prevention guidelines emphasize the importance of identifying the underlying stroke mechanism (eg, small vessel disease, large artery atherosclerosis, cardioembolism), since each mechanism warrants distinct antithrombotic regimens.^136^ In contrast to cardioembolic strokes that necessitate anticoagulation, prevention of strokes related to small or large arteries requires APT. Long-term SAPT, usually with aspirin, is the mainstay secondary prevention strategy for ischemic stroke or TIA.^136^ In contrast, the role of DAPT in early recurrent stroke prevention is time-limited in the acute setting^154–156^ or mechanism-specific^141,156,157^ (eg, intracranial large artery stenosis).

##### Acute ischemic stroke or TIA

Several multicenter RCTs including patients with acute minor ischemic stroke or high-risk TIA have demonstrated a short-term reduction in ischemic stroke recurrence with DAPT-clopidogrel^154,155^ or DAPT-ticagrelor^156^ compared to low-dose aspirin alone. Compared to the aspirin-only group, DAPT-clopidogrel reduced the event rate especially during the first 21 days of therapy (5.2% versus 7.8%; HR 0.66, 95% CI, 0.56-0.77) with a nonsignificant increase in major bleeding (0.3% versus 0.1%; HR 2.11, 95% CI, 0.86-5.17), resulting in a favorable risk-benefit profile (NNT 38; NNH 500).^158^ Accordingly, international stroke guidelines recommend DAPT-clopidogrel for 21 days after noncardioembolic acute minor ischemic stroke or high-risk TIA.^136^

There is no evidence on the effect of TP (with or without cancer) on the risk-benefit ratio in this context. A single retrospective cohort study provided data on TP patients (<100 × 10^9^/L) with acute ischemic stroke (AIS) (n = 28) and demonstrated a high risk of intracranial hemorrhage (14.3% versus 1.5%) compared with patients without TP (n = 1,205), as detailed in **Table 6**.^159^ Although fraught with potential bias, this study provides reason for caution. The risk-benefit ratio in non-TP patients and the time-limited nature of the DAPT (21 d), despite uncertainty and some concern regarding the bleeding risk in TP and cancer, led us to recommending continuing DAPT with clopidogrel for up to 21 days after minor stroke or high-risk TIA in grade 1 TP and in grade 2 TP in the absence of additional bleeding risk factors. Because of a modest NNT, we recommend SAPT (low-dose aspirin) over DAPT in grade 3 TP in this context, and holding all APT in grade 4 TP.

**Recommendations**

**10. AIS or TIA in the past month without a significant intracranial arterial lesion**

**a.** For stable grade 1 TP, DAPT with clopidogrel should be used for 21 days after the event followed by low-dose aspirin only.^136^ ***Level 5, grade D***
**b.** For grade 2 TP, DAPT with clopidogrel should be used for 21 days followed by low-dose aspirin only, unless additional bleeding risk factors are present (**Table 4**). If additional bleeding risk factors are present SAPT with aspirin should be considered instead of DAPT. ***Level 5, grade D***
**c.** For grade 3 TP, SAPT with low-dose aspirin should be used. ***Level 5, grade D***
**d.** For grade 4 TP, We recommend to withhold any APT until platelets reach > 25 × 10^9^/L. ***Level 5, grade D***

##### Symptomatic intracranial large artery stenosis

Determining the stroke mechanism has become a cornerstone of management in recent years, and intracranial large artery stenosis represents one of the leading mechanisms.^136^ Patients with prior ischemic stroke or TIA with a intracranial vessel arterial culprit lesion are at a high risk of recurrent stroke beyond the 21-day time-horizon.^160^ Post hoc analyses of RCT support the use of DAPT with clopidogrel in patients with severe intracranial atherosclerotic stenosis, such as a 12.2% 1-year recurrent stroke rate with DAPT-clopidogrel for 90 days in the medical arm of the SAMMPRIS trial compared with a 25% rate in similar patients from the WASID trial receiving aspirin alone.^143,157,161^ Such analyses have resulted in a 2a recommendation in recent American Heart Association (AHA) stroke guidelines stating that the addition of clopidogrel to aspirin for up to 90 days is reasonable to further reduce recurrent stroke risk in patients with recent AIS or TIA (within 30 d) attributable to severe stenosis (70%–99%) of a major intracranial artery.^136^

Again, TP is not addressed in the above studies and guidelines caused by lack of evidence from RCT. The sizeable absolute risk reduction in non-TP patients and the time-limited nature of the DAPT (90 d), despite uncertainty and some concern regarding the bleeding risk in TP and cancer, led us to recommending continuing DAPT for up to 90 days after recent stroke or TIA attributed to intracranial atherosclerotic stenosis for patients with grade 1–2 TP. Since this is a group with a very-high-risk of recurrent stroke, in patients with grade 3 TP, we recommend SAPT for 21 to 90 days, or DAPT in very specific patients without additional bleeding risk factors. We recommend holding all APT in most patients with grade 4 TP. SAPT with aspirin should be considered if there are stable platelet counts >20,000/µL on an individual basis, in the absence of additional bleeding risk factors (**Table 4**).

Importantly, all decisions on DAPT must be taken after consultation with a vascular neurologist, and the risk of hemorrhagic transformation of AIS should be assessed on a case-by-case basis.

**Recommendations**

**11. Acute symptomatic intracranial arterial stenosis**

**a.** Grade 1–2 TP: we recommend DAPT-clopidogrel for up to 90 days.^136^ ***Level 5, grade D***
**b.** Grade 3 TP: DAPT with clopidogrel for up to 21 days should be used in the absence ofadditional bleeding risk factors. Patients not eligible for DAPT because of additional bleeding risk factors should receive SAPT with aspirin. ***Level 5, grade D***
c. Grade 4 TP: We recommend to withhold DAPT and consider starting SAPT with low-dose aspirin if platelets >25 × 10^9^/ L on an individual basis. ***Level 5, grade D***

#### DAPT in ACS and coronary intervention

Optimal coronary techniques including complete stent apposition using intravascular imaging, adequately sized stents and avoidance of 2-stent bifurcation interventions are of particular importance to prevent subsequent stent thrombosis. In cancer-associated TP, minimizing the intra- and procedure-related bleeding is crucial. The radial access seems associated with approximately halving of short and long-term major bleeding as compared to the femoral access.^162^ In addition, reduced-dose intravenous anticoagulation should be used, with loading doses of unfractionated heparin (UFH) adjusted according to platelet counts as follows: grade 1–2 TP, 50–70 international units (IU)/kg; grade 3–4 TP, 30–50 IU/kg. Activated clotting time (ACT) should be assessed every 30 minutes during intraprocedural anticoagulation, and additional UFH boluses should be given if the ACT is <250 seconds. Furthermore, the upfront combination of UFH and LMWH should be avoided and substituted by fondaparinux 2.5 mg for the indication of non-ST elevation MI (NSTEMI) and 2000 IU UFH should be used for diagnostic transradial cardiac catherization only. Glycoprotein IIb/IIIa inhibitors should be avoided in patients with any degree of TP.^163^ The intravenous P2Y_12_ inhibitor cangrelor may increase the risk of intracranial bleeding and should be avoided in patients with any degree of TP.

Moreover, in stable patients with proof of moderate-to-severe ischemia a recent landmark trial has shown that coronary stent implantation does not reduce the rate of cardiovascular events but relieves symptoms.^164^ This study excluded high-thrombotic risk patients, such as those with left main coronary artery stenosis. To assess the risk associated with coronary disease, coronary angiography should still be considered in cancer TP. The indication for coronary stent implantation should be limited to stenoses influencing the survival of the patient, using the radial approach, if possible, whereas postponing PCI if not urgently necessary until platelet counts are above 100 × 10^9^/L. Factors favoring or discouraging coronary stent implantation in TP patients are detailed in **Table 7**. In patients with ACS who have grade 4 TP or who are anticipated to develop this within the following days, conservative management without PCI is preferred, unless in case of high-risk scenarios (**Table 7**). In case of PCI within 1 month and grade 4 TP, a time-limited strategy (days to 1–2 wks) of platelet transfusion support (targeting platelets > 25 × 10^9^/L) may be considered to enable aspirin SAPT with low-dose aspirin (**Table 6**) in extreme scenarios such as a coronary lesion causing cardiogenic shock, as detailed in recommendation 3.a.ii.

**Table 7.** Factors Favoring or Discouraging Coronary Stent Implantation in Thrombocytopenic Patients^a^

In Favor of Coronary Stent Implantation	Against Coronary Stent Implantation
ACS with one or more of the following: • STEMI especially if presenting early after symptom onset • Life-threatening arrhythmias or hemodynamic instability • Proximal coronary stenosis • Stenosis with features of a culprit lesion	Grade 3 or 4 TP
Coronary stenosis at a site supplying large areas of the myocardium potentially leading to hemodynamic instability, such as left main stenosis or last patent open vessel	Anticipated further drop of platelet count or no recovery within the 4 wks following the planned intervention
Coronary stenosis causing cardiogenic shock	Distalcoronary stenosis or stenosis of a side branch
Availability of intravascular imaging ensuring use of adequately sized stents and optimal stent apposition	Complex coronary lesions as defined in the 2020 ESC guidelines^b^
	Coronary stenosis without an immediate impact on the clinical course based on the lack of features of a culprit lesion and only borderline significance in a noninvasive stress test or measurement of fractional flow reserve
	Alternative diagnoses explaining cardiac symptoms such as uncontrolled hypertensions or valvular heart disease

^a^Defined as patients with existing or anticipated grade 1–4 thrombocytopenia within the following 3 mo.

^b^The 2020 ESC guidelines definition of complex coronary includes but is not limited to the following: coronary stenosis which only can be solved with bifurcation stenting with 2 stents, with very long and small stents, with >3 stents, with >3 stented lesions.^165^

ACS = acute coronary syndrome; STEM = ST elevation MI.

As patients with ACS have the highest risk of early recurrent SVE including stent thrombosis, guidelines recommend 12-month DAPT with one of the potent P2Y_12_ inhibitors prasugrel or ticagrelor over clopidogrel for ACS patients with or without coronary intervention. Phase 3 trials which provided strong evidence for these recommendations excluded patients with TP, with or without cancer, for whom the evidence relies on small and mostly observational cohorts reported in **Table 6**. Recent noninferiority studies have shown that de-escalation to SAPT after a DAPT duration of only 3 months with clopidogrel post-PCI for ACS and after 1 month post-PCI for stable chronic coronary syndrome, is safe, particularly in vulnerable patient groups.^140,165–167^ This strategy may also be adopted in patients with TP, in whom the indication for DAPT should be re-evaluated when TP occurs as well. Thus, in the context of TP, one should consider the relatively lower bleeding risk associated with DAPT with clopidogrel as compared to DAPT with ticagrelor or prasugrel.^168^ Moreover, extended (eg, >12 mo) DAPT should be avoided in patients with any degree of TP and cancer caused by an elevated bleeding risk.

Specifically in TP patients with or without cancer, the limited evidence, reported in **Table 6**, can be summarized as follows. There was no major bleeding among 98 cancer patients with chronic TP (<100 × 10^9^/L; 37% <30 × 10^9^/L) undergoing PCI for ACS, of whom 42% received aspirin and 28% received both aspirin and clopidogrel.^163^ In this cohort, CV mortality was higher in grade 4 TP, whereas statins and aspirin alone or in combination with clopidogrel were associated with a trend toward longer survival (*P* = 0.06). Similarly, among 119 patients with acute MI, cancer and TP (61 with platelets <50 × 10^9^/L) aspirin was associated with a higher survival at 7 days, 1 and 3 year as compared to patients not receiving aspirin, with no differences in bleeding.^20^ Similar data were reported by Sarkiss et al, on 47 ACS patients with a TP averaging 32 × 10^9^/L, where the lack of aspirin treatment was associated with a higher 7-day death.^169^

In patients with stable angina in whom the ESC guidelines recommend 6 months of DAPT with clopidogrel after drug-eluting stent implantation,^135^ the duration of DAPT may be reduced to 1 month in patients at very-high risk of bleeding.^170–172^ Therefore, one may consider changing DAPT to SAPT with aspirin in stable high degree TP patients with coronary stents 1 month or later after the procedure.

**Figure 3.**
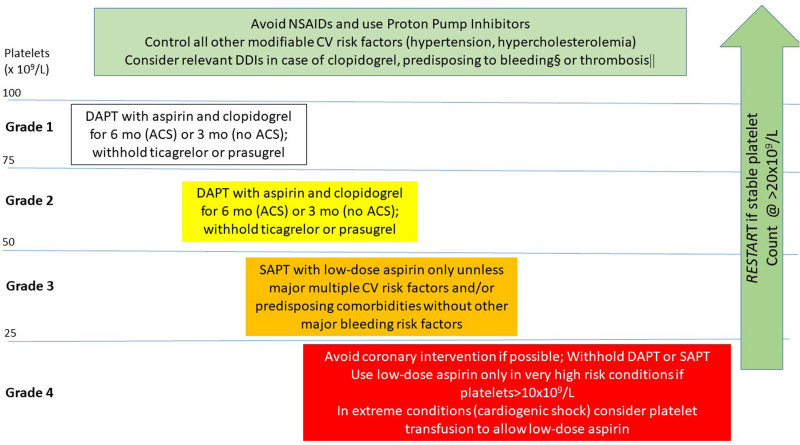
**Management of APT in patients with ACS and POST-PCI.** §Refer to **Table 4** for a nonexhaustive list of risk factors for major bleeding. ||Refer to **Table 3** for a nonexhaustive list of patients with high-thrombotic risk. ACS = acute coronary syndrome; CV = cardiovascular; DDI = drug-drug interaction; DAPT = dual antiplatelet therapy; NSAID = nonsteroidal anti-inflammatory drugs; SAPT = single antiplatelet therapy; TP = thrombocytopenia.

In patients with TP and PCI caused by stable angina in the previous month, or ACS in the previous 3 months, we propose the following adjustments: avoid ticagrelor and prasugrel with any grade of TP, except for specific very-high-risk thrombotic scenarios (**Table 3**) in patients with stable platelet counts and grade 1 TP only; consider SAPT with aspirin for grade 3 TP; withdraw SAPT for grade 4 TP. In case of early de-escalation from DAPT to SAPT within the first 4 wks post-PCI, this should be performed in a tertiary center with close monitoring of electrocardiogram and cardiac enzymes and 24-hour availability of coronary interventions as well as a predefined strategy of platelet inhibition in case of a recurrent cardiac event such as ACS or stent thrombosis. **Figure 3** provides an overview on antiplatelet therapy management in patients with ACS and POST-PCI.

**Recommendations**

**12. APT management in ACS and POST-PCI**

**a.** Grade 1–4 TP: IV antiplatelet drugs (GPI, P2Y_12_ inhibitors) are not recommended. ***Level 5, grade D***
**b.** Grade 1 TP: We recommend DAPT with clopidogrel in patients with ACS within the previous 12 months, unless high-risk thrombotic factors (**Table 3**) are present in the absence of major additional bleeding risk factors (**Table 4**).^173^ ***Level 3b, grade C***
**c.** Grade 2–4 TP: We recommend against DAPT with ticagrelor or prasugrel. ***Level 5, grade D***
**d.** Grade 2: We recommend using DAPT with clopidogrel. ***Level 3b, grade C***
**e.** Grade 1–2 TP: Consider shortening the duration of DAPT and changing to SAPT after PCI with a modern drug-eluting stent^172,174-176^ to 6 months after ACS and to 3 months if no recent ACS. An individualized decision in consensus with the treating interventional cardiologist is needed. ***Level 5, grade D***
f. Grade 3 TP: We recommend SAPT with low-dose aspirin in all nonhigh-thrombotic risk settings. Consider DAPT with clopidogrel only in very-high-thrombotic risk events occurring in the prior 3 months and avoid coronary intervention if possible*. ***Level 3b, grade C***
**g.** Grade 4 TP: DAPT and SAPT should be withheld, except in case of very-high-thrombotic risk events that may justify SAPT with low-dose aspirin at platelet counts above 10 × 10^9^/L. Coronary intervention should be avoided. ***Level 3b, grade C***

*ACS with a culprit lesion in a proximal coronary vessel or in patients hemodynamically compromised including therapy-refractory ventricular arrhythmias or bradycardias, coronary stenosis at a site supplying large areas of the myocardium potentially leading to hemodynamic instability such as left main stenosis or last patent open vessel, cardiogenic shock caused by a coronary stenosis.

## COMBINED APT AND OAC

### APT and full-dose OAC

The majority of data stems from randomized clinical trials in patients with AF with acute indications for APT because of concomitant ACS or revascularization. The AUGUSTUS trial demonstrated that among patients with AF and ACS or PCI treated with a P2Y_12_ inhibitor and oral anticoagulation (VKA or apixaban), the 6-month incidence of major or clinically relevant nonmajor bleeding was higher among patients receiving DAPT than in those treated with clopidogrel and placebo, and was higher in the group of VKA versus DOAC.^177^ Of note, a meta-analysis of these studies showed a significant increase in stent thrombosis and increased MI in patients not receiving aspirin.^178^ A post hoc analysis of AUGUSTUS showed that 80% of stent thrombosis occurred within the first 30 days and all of them had severe clinical consequences (recurrent MI, death). There is no data on management of patients receiving anticoagulation for VTE who have ACS or undergo PCI. Since VTE (especially within 3 mo) has a short-term thrombotic risk that is often at least comparable to that of AF, and because the management strategies maintain the anticoagulation component, we propose extrapolating the AF data to patients with VTE in the past 3 months.

Therefore, in case of stable grade 1 and 2 TP, we recommend combined SAPT with clopidogrel with a DOAC (rather than DAPT and VKA) in patients with any indication for OAC (eg, AF or VTE) from 1 month after ACS or 1 wk from coronary revascularization for CCS. The rationale is that the degree of TP would tip the balance toward bleeding, thus preferring a regimen with a lower bleeding risk for higher degrees of TP. When SAPT-clopidogrel and DOAC is continued in patients with stable grade 1 TP, the lowest effective anticoagulant dose should be used, unless the patient has a low bleeding risk. In this respect, the 2020 ESC AF guidelines suggest reduced-dose rivaroxaban (15 mg od) or dabigatran (110 mg bid) for high-bleeding risk patients in case of combined antithrombotic therapy.^59^ In patients with grade 3–4 TP, AF, and recent ACS, we recommend withdrawing DOAC and continuing with SAPT with low-dose aspirin.^179^

In case of patients with AF and chronic CAD or revascularization >1 year earlier, we recommend in patients with grade 1–2 TP receiving OAC (for any indication) and in highly selected patients with grade 3 TP receiving any anticoagulation (see recommendation 5.b) that SAPT should be generally avoided in case of nonacute, chronic indications such as PCI or coronary artery bypass graft (CABG) >1 year earlier or CAD not requiring revascularization.

**Recommendations**

**13. Management of combined APT and OAC**

**a.** Grade 1–2 TP: In stable grade 1 TP, combined SAPT and OAC should be considered in patients with AF or VTE within 3 months from 1 month after ACS or 1 wk after coronary revascularization for CCS. ***Level 5, grade D***
**b.** Grade 1–2 TP: In patients receiving OAC for AF or post-VTE, and SAPT for a nonacute, chronic indication such as PCI or CABG >1 year earlier or CAD not requiring revascularization, SAPT should be withdrawn and OAC should be managed according to the indication as detailed in recommendation. ***Level 5, grade D***
**c.** Grade 3 TP: Combined SAPT and OAC are not recommended and patients should maintain SAPT with low-dose aspirin. ***Level 5, grade D***
d. Grade 4 TP: Combined SAPT and OAC are not recommended. SAPT with low-dose aspirin may be considered in very-high-thrombotic risk events and platelet counts above 10 × 10^9^/L. ***Level 5, grade D***
e. Grade 4 TP:We recommend against coronary intervention. ***Level 5, grade D***

### Aspirin and very low-dose DOAC

Stable CAD patients with previous MI or symptomatic PAD and additional CV risk factors (eg, age >65 y, diabetes, smoking habits, estimated glomerular filtration rate <60 mL/min, heart failure, or nonlacunar ischemic stroke ≥1 mo earlier) have been shown to benefit from the combination of low-dose aspirin and very low-dose rivaroxaban (2.5 mg bid), also known as dual pathway inhibition.^180^ However, since the bleeding risk was significantly increased by this combination,^180^ (NNT 79 versus NNH 81) and in the absence of any evidence in TP, we advise to withhold this combination of aspirin and rivaroxaban for all TP grades and adjust aspirin as detailed in recommendation no. 8.

**Recommendations**

**14. Management of dual pathway inhibition**

**a.** For grade 1–4 TP: We recommend against very low-dose rivaroxaban in association with aspirin at any degree of TP. ***Level 5, grade D***
**b.** For grade 1–4 TP: We recommend continuing with low-dose aspirin alone according with recommendations for SAPT and secondary prevention (see recommendation 8). ***Level 5, grade D***

## REPERFUSION THERAPY

### Reperfusion strategies for patients with AIS and TP

#### Thrombolytic therapy for patients with AIS and TP

For patients with AIS and a platelet count below 100 × 10^9^/L, intravenous thrombolysis using recombinant tissue plasminogen activator (rt-PA, alteplase) or tenecteplase is contraindicated according to the European Stroke Organization (ESO) and the AHA/American Stroke Association (ASA) guidelines.^181,182^ However, according to the most recent updated recommendations, for patients with AIS within <4.5 hours of symptom onset and with unknown platelet count, intravenous thrombolysis is suggested to be initiated as soon as possible and should not be delayed by waiting for the results of laboratory tests, unless there is a strong reason (available detailed medical record) to expect an abnormal platelet count. In any case, if the platelet count is found to be lower than 100 × 10^9^/L, intravenous thrombolysis should not be administered, and initiated treatment must be discontinued.^182^ As the history of cancer associated with TP might not be an immediately available information at stroke presentation, caution must be taken when the family members or the patient are unable to provide a clear and complete medical history. Even in such cases, efforts to reduce laboratory delays are of outmost importance in AIS care. Rapid laboratory results of platelet count generally allow thrombolysis to be discontinued promptly even after the initiation of treatment in patients subsequently proven to have TP.^183^

**Figure 4.**
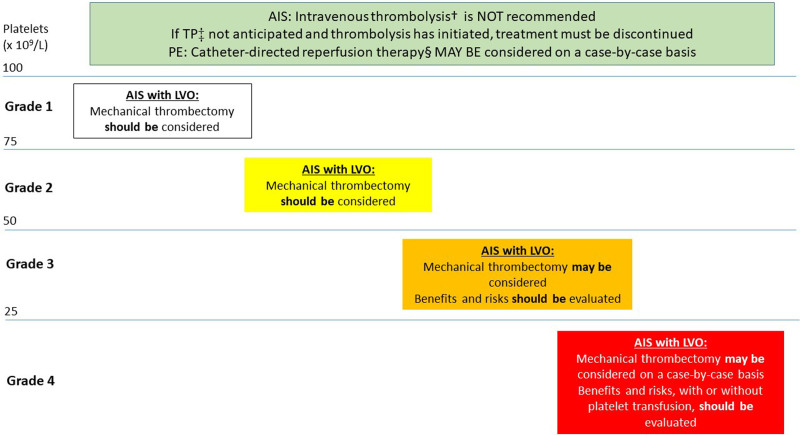
**Reperfusion therapy in cancer patients with TP‡.** †Recombinant tissue plasminogen activator (rt-PA, alteplase) or tenecteplase. ‡Platelets <100 × 10^9^/L. §Interventional procedures of thrombus removal including catheter-based thrombolysis or pharmacomechanical catheter-directed reperfusion techniques. AIS = acute ischemic stroke; LVO = large vessel occlusion; PE = pulmonary embolism; TP = thrombocytopenia.

Currently, the safety and efficacy of thrombolysis are unknown in TP cancer patients, as TP was an exclusion criteria in almost all RCT of intravenous thrombolysis in AIS patients.^181^ In trials where such patients were not excluded (eg, ECASS I, IST-3), subgroup analyses of the TP subgroup are not published.^184,185^ Available evidence in patients with TP receiving intravenous rt-PA suggests that low platelet count is associated with a significantly higher risk of symptomatic intracranial hemorrhage (sICH). In a prospective, multicenter, observational study from 10 European centers including 7533 patients, 595 (7.9%) patients had platelet count <150 × 10^9^/L, whereas only 44 patients (0.6%) had TP <100 × 10^9^/L.^186^ Low platelet count was found to be associated with a significantly higher risk of sICH in a multivariable analysis (adjusted OR 1.73; 95% CI, 1.24–2.43; *P* < 0.002) but was not associated with poor functional outcome at 3 months postevent according to the modified Rankin Scale or death. Conversely, TP was not significantly associated with sICH in an unadjusted model, most probably because of the relatively small sample size. In another large retrospective study using the 2012–2014 National Inpatient Sample of US hospitals, among 101,527 patients treated with intravenous rt-PA, 3520 (3,47%) were identified with TP.^187^ In a multivariate analysis, TP was associated with a significantly higher incidence of intracranial hemorrhage (adjusted OR 1.82; 95% CI, 1.37-2.42; *P* < 0.001), postprocedural bleeding, higher in-hospital mortality, longer length of stay, higher incidence of tracheotomy and mechanical ventilation. **Figure 4** provides an overview on reperfusion therapy in cancer patients with TP.

#### Mechanical thrombectomy for patients with AIS and TP

In AIS patients with TP and large vessel occlusion (LVO), interventional thrombectomy without pharmacological agents is a potentially safer option. As compared to intravenous thrombolysis, considerably lower thresholds of platelet counts were used as exclusion criteria in a handful of interventional thrombectomy studies, such as 30 × 10^9^/L in the Multi-MERCİ and 40 × 10^9^/L in the MR CLEAN study.^188,189^ Case reports have been published with even lower thresholds for attempting recanalization by mechanical thrombectomy in the setting of AIS patients with LVO.^190^ Limited data based on the pooled analysis of the MERCI and MULTI-MERCI trials (n = 6 patients) raised no safety concerns using the relatively low threshold platelet count of 30 × 10^9^/L because only one case of sICH was detected in these studies, and that particular patient was treated outside protocol (platelet count 16 × 10^9^/L).^191^ On the other hand, it must be noted that despite successful recanalization in the majority of patients with low platelet count (4/6), none of the studied patients showed favorable functional outcomes and most patients died (modified Rankin Scale: 4 in 2 patients and death in 4 patients). This was most likely related to baseline comorbidities, including advanced malignancies likely influencing outcomes. Similarly, other retrospective analyses on the rates of sICH in TP AIS patients undergoing mechanical thrombectomy did not identify major differences in the incidence of sICH among patients with or without TP.^192,193^ On the other hand, worse functional outcomes and higher mortality were found in theTP group, most probably because of worse prestroke functional status and pre-existing morbidity.

Accordingly, a most recent report of the Society of Neuro Interventional Surgery Standards and Guidelines Committee proposes that mechanical thrombectomy should not be withheld from TP patients and they define no absolute minimum platelet count that would exclude patients from mechanical thrombectomy.^196^ Given the high morbidity of LVO, mechanical thrombectomy should definitely be considered for patients with grade 1 and 2 TP. Grade 3 TP might be a concern for intracerebral hemorrhage or procedural bleeding complication, particularly in cancer patients, and according to this expert panel, individualized treatment decisions should be made based on the evaluation of the risk/benefit ratio of the procedure (potential risk of bleeding versus mortality). Grade 4 TP is considered at very-high risk for hemorrhagic or procedural complications. In patients with grade 4 TP, some experts consider platelet transfusion to be reasonable to avoid bleeding complications during the procedure.^196^

**Recommendations**

**15. Reperfusion therapy for patients with AIS**

a. Grade 1–4 TP: In patients with AIS, we recommend against intravenous thrombolysis because of unknown safety and potentially increased risk of major hemorrhagic complications. ***Level 4, grade C***
b. If TP is not anticipated and treatment was initiated, thrombolysis must be discontinued upon documented TP. ***Level 4, grade C***
c. Grade 1–2 TP: In patients with acute ischemic stroke caused by LVO, an endovascular mechanical thrombectomy should be considered in all eligible patients. ***Level 2b, grade C***
d. Grade 3 TP: In patients with AIS caused by LVO, an endovascular treatment strategy (mechanical thrombectomy) may be considered based on the individual patient’s thrombotic and bleeding risks. ***Level 5, grade D***
**e.** Grade 4 TP: Based on the very-high associated bleeding risk, benefits and risks of mechanical thrombectomy, with or without platelet transfusion, should be evaluated on a case-by-case basis. ***Level 5, grade D***

### Systemic Thrombolytic Therapy and Complex Reperfusion Strategies for Patients With tp and pe

Based on current guidelines, since cancer patients have a higher incidence of VTE but also a higher incidence of bleeding, malignancy is a relative contraindication to systemic (intravenous) thrombolysis in VTE.^126,195,196^ Accordingly, data for thrombolysis in patients with TP and cancer are scarce.^197^ Based on the high risk of bleeding in case of systemic thrombolysis, this therapy in patients with TP and cancer should be avoided or considered only on a case-by-case basis in high-risk patients with massive PE (with persistent arterial hypotension or shock) and only in centers with appropriate expertise. It must be emphasized that full-dose intravenous thrombolysis can be associated with life-threatening bleeding complications, particularly intracranial hemorrhage, even in the absence of TP.^199^

In patients with immediate-high risk or submassive PE, catheter-derived thrombolysis or pharmacomechanical catheter-directed reperfusion techniques might be considered as more promising and safer options.^199^ Nevertheless, consultation with a specialist experienced in catheter-derived thrombolysis is advised in all cases and procedures should only be performed in specialized centers with highly trained personnel. During these interventions, selective infusion of the thrombolytic agent is often combined with various interventional techniques such as thrombus aspiration, destruction, or ultrasound emission.

Ultrasound assisted catheter-directed thrombolysis is currently the most studied catheter-derived pharmacomechanical technique.^200–204^ Catheter-directed approaches allow the use of a considerably lower dose of thrombolytics (~1/10 as compared to systemic treatment), thus these type of procedures are possibly associated with a lower risk of bleeding.^198,203,205^ However, data for this therapy is still evolving and it cannot be used in high-risk PE and severely compromised patients, unless intravenous thrombolysis is contraindicated or failed. In high-risk patients with contraindications for systemic treatment, although guidelines may advocate the use of catheter-directed thrombolysis, caution is warranted in patient selection because of the high incidence of major bleeding and mortality in this population.^196,199,203–205^ Another limitation is that the handling of this system requires expertise in navigating in the pulmonary arteries, which suits only the most experienced interventional centers. It must be emphasized that currently any form of thrombolysis in patients with PE and TP remains a challenge caused by the lack of evidence, as mostly case reports are available in the literature.^197^ Unfortunately, based on current low evidence level data, a platelet count threshold in cancer patients for safely carrying out these interventions without significant bleeding risk cannot be provided, as yet, and patients should be evaluated on a case-by-case basis.

**Recommendations**

**16. Reperfusion therapy for patients with PE**

a. For grade 1–4 TP: we recommend against systemic thrombolysis in VTE patients caused by unknown efficacy and potentially high-bleeding risk. ***Level 5, grade D***
b. For grade 1–3 TP: patients with high-risk PE and in centers with appropriate expertise systemic thrombolysis may be considered on a case-by-case basis. ***Level 5, grade D***
c. For grade 1–4 TP: interventional procedures of thrombus removal including catheter-based thrombolysis or pharmacomechanical catheter-directed reperfusion techniques may be considered on a case-by-case basis with a specialist owing adequate interventional expertise of centers with appropriate experience. ***Level 5, grade D***

## CONCLUSIONS

As the overall cancer death rate has declined over the past decades and the cancer survival has increased caused by improved medicines and management, nevertheless new, unforeseen challenges are emerging. Cancer and cardiovascular disease are increasingly sharing the same risk factors (diabetes, obesity, smoking) and some pathogenic mechanisms of complications, including thrombotic events. The long survival and new medicines are progressively raising the incidence of VTE and atherothrombotic diseases in cancer patients, even more than the general population. TP which is often associated with cancer disease or anticancer therapy, will be more and more present in acute or stable thrombotic cancer patients, but evidence on how-to-treat these patients is lacking caused by the exclusion of cancer or TP patients from cardiovascular RCT. These trends show that progress is being made against the disease but much work remains. Data on anticoagulation dose reduction in acute VTE and grade 1–4 TP look promising^67^; however, there is an unmet need for evidence on anticoagulation dose reduction in patients with AF and grade 3–4 TP, especially in patients with a high-thrombotic risk.

Thus, how-to-treat TP cancer patients with acute or previous atherothrombotic or thromboembolic events remains a substantial unmet therapeutic need, a huge gap in knowledge and a challenge for the very near future. Clinical studies in this field are highly needed; however, observational studies (preferably multinational registries), mechanistic, proof-of-concept, and even *in silico* studies are central to design clinical studies in this special and unique patient population, with a tight balance between thrombosis and bleeding. Possible future directions for research in cancer patients with grade 3–4 TP receiving antithrombotic therapy include: clinical trials assessing the proper anticoagulation dose reduction as compared to full dose, the efficacy of platelet transfusion in acute VTE and grade 3–4 TP; whether dose reduction is superior to no anticoagulation in AF patients with grade 3 TP for a limited time frame; observational studies on safety of systemic thrombolytic therapy and complex reperfusion strategies in cancer patients with PE or AIS and various TP degrees, the medical versus revascularization management in ACS as a function of platelet counts below the exclusion criteria of traditional RCTs. International collaboration will be crucial in achieving these urgent therapeutic goals.

## ACKNOWLEDGMENTS

We wish to acknowledge Dr Rani Barnea (Vascular Neurologist, Stroke Unit, Rabin Medical Center, Petah Tikva, Israel) for his work and input on the neurovascular aspects of the manuscript; Dr Cinzia Giaccherini (Hospital Papa Giovanni XXIII, Bergamo Italy) for revising and editing the manuscript. **EHA representatives:** Falanga A, Leader A, Ambaglio C, Bagoly Z, Castaman G, Elalamy I, Lecumberri R, Pabinger I, Trinchero A, Ten Cate H. **ESC representatives:** Niessner A, Szmit S, Rocca B.

## AUTHOR CONTRIBUTIONS

Chair and coordinator: AF. Steering Committee: IP, IE, GC. Panel members: AL, CA, ZB, RL, AN, SS, AT, HTC, BR. All the authors searched the literature, extracted data from eligible studies, analyzed the data, prepared evidence summaries and wrote the manuscript. Specifically, AF, AL, CA, GC, IE, RL contributed to anticoagulant therapy section specifically; AF, AL, AN, AT, BR, SS, HTC contributed to antiplatelet therapy section; AF, BZ, AN contributed to reperfusion therapy section. All authors assessed the evidence, voted, made judgments and contributed to critical revisions of the whole manuscript.

## DISCLOSURES

AF receives royalties of payments from Stago, Werfen, Sanofi and Bayer. AF receives royalties of payments from Bayer, Novartis, Pfizer and Sanofi. GC receives royalties of payments from Grifols, Roche, Novo Nordisk, and Sobi. IE receives royalties of payments from Sanofi, Bristol Myers Squibb, Pfizer, Leo-Pharma, Aspen, Boehringer Ingelheim, Bayer and Stago. RL receives royalties of payments from Laboratories ROVI, Leo-Pharma, BMS, and Sanofi. AN receives royalties of payments from Astra Zeneca, Bayer, Bristol Myers Squibb, Boehringer Ingelheim, Daichi Sankyo, and Pfizer. IP receives royalties of payments from Novartis, Amgen, CSL Behring, NovoNordisk, Bayer, Takeda, Pfizer, Biotest, and Sobi. SS receives royalties of payments from Amgen, Angelini, AstraZeneca, Bayer, Bristol Myers Squibb, Pfizer and Teva. HTC receives research support from Bayer, receives consulting fees from Alveron and is a stock holder from Coagulation Profile. BR receives consulting fees from Aboca Societa’ Agricola srl, research funding from Bayer AG, and lecture fees from Sobi. The other authors report no conflict of interest.

## Supplementary Material


