# Supplementary material for: EHA Guidelines on Management of Antithrombotic Treatments in Thrombocytopenic Patients With Cancer
Source: Hemasphere. 2022 Jul 13;6(8):e750. doi: 10.1097/HS9.0000000000000750 (PMC9281983; doi:10.1097/HS9.0000000000000750)
Supplement: Supplementary file 1 [file hs9-6-e750-s001.docx]

**Supplemental material**

- **Appendix 1. Keyword list**
- **Appendix 2. Panelists and Delphi consensus**
- **Supplemental table 1**

**Appendix 1. Keyword list**

Acute coronary syndrome, anticoagulation, anticoagulants, antiplatelet, aspirin, apixaban, atrial fibrillation, bleeding, cancer, chronic coronary syndrome, coronary reperfusion, clopidogrel, device-related thrombosis, dabigatran, DAPT, DOAC, edoxaban, heparin, haematological malignancies, leaf atrial appendage occlusion, leukemia, low-molecular weight heparin, myocardial infarction, neoplasms, platelet aggregation inhibitors , PCI, PAD, rivaroxaban, thrombocytopenia, thrombocytopenic, thrombolytic therapy or thrombectomy, thrombosis, thromboembolism, single antiplatelet therapy, stroke, TIA, unfractionated heparin, vitamin K antagonist, warfarin.

**Appendix 2. Panelists and Delphi consensus**

**Composition of panel membership**

**Expertise**

The EHA and ESC agreed in advance the proposed composition of the Task Force and Delphi consensus panel with the following expertise among Panelists which included:

- Onco-Hematologist
- Cardiologist
- Cardiovascular Pharmacologist
- Internist
- Vascular medicine specialist
- Vascular Surgeon
- Experts in clinical thrombosis and hemostasis
- Experts in laboratory coagulation

**Panel members**

Falanga A

Leader A

Ambaglio C

Bagoly Z

Castaman G

Elalamy I

Lecumberri R

Niessner A

Pabinger I

Szmit S

Trinchero A

Ten Cate H

Rocca B

**Consensus of opinion development**

Each section of the document was first assigned to 3-4 Experts in the specific field (one hematologist and 2-3 other experts chosen based on their expertise for the specific chapter). Then for each section the assigned Panelists drafted a first proposal of consensus statements, based on the available evidence.

A Delphi method of consensus development(1) was then used, and included three survey rounds.

In Round 1, participants were asked to rate agreement/disagreement with initial statements across each section. Participants were also asked to contribute further ideas in relation to these topics, which were incorporated as new statements in Round 2. Comments from Round 1 were analyzed and categorized by theme and considered when developing the recommendation (for accepted statements) or amending the statement for inclusion in Round 2 (for non-accepted statements).

In Rounds 2 and 3 participants reappraised their ratings in view of the group consensus.

All Experts (13/13) completed Round 1 and gave their responses, 11/13 completed Round 2 (85%) and 12/13 completed Round 3 (92%).

Consensus (defined as > 70% agreement) was achieved for all the recommendation statements, with 100% consensus on Round 3.

**Reference**

1. Rowe G, Wright G. The Delphi technique as a forecasting tool: issues and analysis. Int J Forecasting 1999;15:353-375.

**Supplemental Table 1.** Summary of clinically relevant DDIs between antithrombotic drugs and drugs currently used in cancer patients

| **Drug Combinations** | **Relevant mechanism of action** † | **Haemostatic clinical effect** ‡ | **Possible effect on the anti-cancer drug** | **Strength of interaction & advice** § |
| --- | --- | --- | --- | --- |
| **ANTIPLATELET DRUGS** | | | | |
| ***Clopidogrel plus…*** | Prodrug converted to its active metabolite via CYPs 3A4, 2B6, 2C19, 1A2. Inhibitor of CYP2C8. |  | | |
| ***Enzalutamide*** | Strong inducer of CYP3A4, moderate inducer of CYP2C19. | Concentrations of clopidogrel active metabolites may increase and a supratherapeutic effect may occur.  **⭡ risk of bleeding** | If the combination cannot be avoided, a dose reduction of enzalutamide  from once daily should be considered, based on careful monitoring of tolerability. | **Strong interaction:**  **AVOID the combination**  Alternative antiplatelet, with no or minimal potential to interact should be considered. Low dose aspirin can be an alternative in case of SAPT due to lack of CYP450-mediated metabolism. If DAPT is necessary, may consider using reduced dose prasugrel. |
| ***Idelalisib*** | Primary metabolite of idelalisib (GS-563117): strong inhibitor of CYP3A4 | Significantly decreased concentrations of clopidogrel active metabolite. Reduced platelet inhibition  **⭡ risk of thrombosis** | None | **Strong interaction:**  **AVOID the combination**  Alternative antiplatelet, with no or minimal potential to interact should be considered. Low dose aspirin can be an alternative in case of SAPT due to lack of CYP450-mediated metabolism. |
| ***Imatinib*** | Moderate inhibitor of the CYP3A4. | Decreased concentrations  of the clopidogrel active metabolite.  Reduced platelet inhibition.  **⭡ risk of thrombosis** | None | **Moderate interaction:**  **CAUTION**  Alternative antiplatelet, with no or minimal potential to interact may be considered. Low dose aspirin can be an alternative in case of SAPT due to lack of CYP450-mediated metabolism. |
| ***Paclitaxel*** | Clopidogrel is an inhibitor of CYP2C8. Thus, the biotransformation of Paclitaxel (which is dependant on CYP2C8) is decreased | None | Paclitaxel concentrations may increase. Clopidogrel with high-dose paclitaxel is associated with an increased risk of neuropathy. | **Moderate interaction:**  **CAUTION**  If coadministration is unavoidable, monitor closely for paclitaxel toxicity |
| ***Ticagrelor plus….*** | Metabolised by CYP3A4 and is substrate of P-gp. |  | | |
| ***Enzalutamide*** | Strong inducer of CYP3A4 | Significantly decreased concentrations of ticagrelor. Possible reduced platelet inhibition. Higher variability in effectiveness  **⭡ risk of thrombosis** | None | **Strong interaction:**  **AVOID coadministration**  Alternate antiplatelet with no or minimal potential to interact with CYP3A4 should be considered.  Prasugrel can be an option in case of DAPT based on lack of DDI with enzalutamide. |
| ***Idelalisib*** | Primary metabolite of idelalisib (GS-563117): strong inhibitor of CYP3A4. | Significantly decreased concentrations of ticagrelor. Possible reduced platelet inhibition. Higher variability in effectiveness.  **⭡ risk of thrombosis** | None | **Strong interaction:**  **AVOID coadministration**  Alternate antiplatelet with no or minimal potential to interact with CYP3A4 should be considered. Prasugrel can be an option in case of DAPT based on lack of DDI with enzalutamide. |
| **ANTICOAGULANT DRUGS** | | | | |
| ***Warfarin plus …*** | R-warfarin: primarily metabolised by CYPs 1A2 and 3A4. S-warfarin: metabolised by CYP2C9. |  | | |
| ***Imatinib*** | Moderate inhibitor of the CYP3A4 and associated per se with bleeding risk | Increased warfarin concentrations.  **⭡ risk of bleeding** | None | **Strong interaction:**  **CONTRAINDICATED**  Consider DOAC (dabigatran, edoxaban) or LMWH as alternatives to warfarin, or monitor INR closely if warfarin is necessary. |
| ***Enzalutamide*** | Strong inducer of CYP3A4; moderate inducer of CYP2C9. | Decreased warfarin concentrations (AUC reduced by 56% in healthy subjects). Higher variability. After discontinuation of enzalutamide, the effect of the interaction can persist for up to several weeks.  **⭡ risk of thrombosis** | None | **Strong interaction:**  **AVOID if possible**  Consider DOAC (edoxaban) or LMWH as alternatives to warfarin, or monitor INR closely if warfarin is necessary. |
| ***Sotorasib*** | Time-dependent inhibitor and inducer of CYP3A. | According to the US drug label, warfarin concentrations may decrease  **⭡ risk of thrombosis** | None | **Strong interaction:**  **AVOID if possible**  Consider LMWH as an alternative to warfarin, or continue warfarin and monitor INR more often |
| ***Idelalisib*** | Primary metabolite of idelalisib (GS-563117): strong inhibitor of CYP3A4 | May significantly increase concentrations of warfarin  **⭡ risk of bleeding** | None | **Strong interaction:**  **AVOID if possible**  Consider DOAC (dabigatran, edoxaban) or LMWH as an alternative to warfarin, or  continue warfarin and monitor INR more often, even following cessation of idelalisib. |
| ***Dabrafenib*** | Inducer of CYP3A4 and CYP2C9. | In patients with BRAF V600 positive tumors, coadministration of warfarin (single dose of 15 mg) decreased S- and R- warfarin AUC by 37% and 33%, respectively.  **⭡ risk of thrombosis** | Unlikely | **Moderated interaction:**  **CAUTION**  Consider DOAC (dabigatran, edoxaban, rivaroxaban) or LMWH as an alternative to warfarin, or continue warfarin and monitor INR closely |
| ***Capecitabine*** | Moderate-to-strong inhibitor of CYP2C9 | Increased concentrations of  warfarin, enhanced anticoagulant activity. In patients with cancer, coadministration of capecitabine and warfarin increased S-warfarin AUC by 57% but not Cmax.  **⭡ risk of bleeding** | None | **Moderate interaction:**  **CAUTION**  Consider DOAC (all) or LMWH as an alternative to warfarin, or continue warfarin and monitor INR closely |
| ***5-fluorouracile*** | May inhibit CYP2C9 | Increase concentrations of warfarin.  **⭡ risk of bleeding** | None | **Moderate interaction:**  **CAUTION**  Closely monitor INR |
| ***Sunitinib***  ***Sorafenib*** | No effect on CYP-450s | **⭡ risk of bleeding** | None | **CAUTION**  Monitor bleeding symptoms |
| ***Dabigatran plus…*** | Substrate of P-gp |  | | |
| ***Olaparib***  ***Lapatinib***  ***Sotorasib*** | P-gp inhibitors | Possible increased dabigatran concentrations and consequent high inter-individual variability  **⭡ risk of bleeding** | Unlikely | **Moderate interaction:**  **CAUTION**  Check for bleeding signs. Consider an alternative DOAC (apixaban, rivaroxaban). Measure ECT or dTT if co-administration cannot be avoided, in case of bleeding or high-degree TP, if available. |
| ***Enzalutamide*** | Inhibitor for P-gp in vitro. | May increase dabigatran concentrations. The clinical relevance of this interaction is unknown,  Higher variability. After discontinuation of enzalutamide, the effect of the interaction can persist for up to several weeks.  **⭡ risk of bleeding** | None | **Moderate interaction:**  **CAUTION**  If possible, an alternate DOAC should be considered (edoxaban). If coadministration is necessary, measure ECT, dTT is recommended. |
| ***Venetoclax*** | May inhibit intestinal P-gp | Possible increased dabigatran plasma concentrations and consequent high inter-individual variability  **⭡ risk of bleeding** | Unlikely | **Moderate interaction:**  **CAUTION**  Check for bleeding signs. Consider an alternative DOAC (apixaban) . Measure ECT or dTT if available and necessary (bleeding, high degree TP). |
| ***Edoxaban plus…*** | Partially metabolised by CYP3A4 (<10%) and substrate of P-gp |  | | |
| ***Sotorasib*** | Time-dependent  inhibitor and inducer of CYP3A, inhibitor of P-gp. | Unknown what the combined effect of P-gp inhibition and CYP3A induction on edoxaban exposure. Increased variability in response | Unlikely | **Moderate interaction:**  **CAUTION**  Consider LMWH instead. If coadministration is unavoidable, monitor closely for edoxaban toxicity and efficacy and measure anti-Xa activity, if available. |
| ***Olaparib***  ***Lapatinib***  ***Neratinib*** | P-gp inhibitors and increase the bleeding risk per se. | Possible increased edoxaban concentrations.  **⭡ risk of bleeding** | Unlikely | **Moderate interaction:**  **CAUTION**  Consider warfarin or LMWHs as alternatives. If coadministration is unavoidable, monitor closely for edoxaban toxicity and measure anti-Xa activity, if available. |
| ***Apixaban plus ….*** | Metabolised by CYP3A4 and to a lesser extent by CYPs 1A2, 2C8, 2C9, 2C19. Substrate for P-gp and BCRP. |  | | |
| ***Enzalutamide*** | Strong inducer of CYP3A4; moderate inducer of CYP2C9 and 2C19; weak inducer of 1A2 | Concentrations of apixaban may significantly decrease. High variability in response.  **⭡ risk of thrombosis** | Unlikely | **Strong interaction:**  **AVOID**  Consider an alternative DOAC (edoxaban) or LMWHs. If coadministration is unavoidable, monitor closely anti-Xa activity, if available. |
| ***Idelalisib*** | Primary metabolite of idelalisib (GS-563117): strong inhibitor of CYP3A4 | May significantly increase concentrations of apixaban.  **⭡ risk of bleeding** | None | **Strong interaction:**  **AVOID if possible**  Consider alternative DOAC (dabigatran, edoxaban). If coadministration is unavoidable, monitor closely anti-Xa activity, if available. |
| ***Sotorasib*** | Time-dependent  inhibitor and inducer of CYP3A | Concentrations of apixaban may decrease  **⭡ risk of thrombosis** | None | **Strong interaction:**  **AVOID if possible**  Consider LMWHs instead. If coadministration is unavoidable, monitor closely for anti-Xa activity, if available. |
| ***Dabrafenib*** | Inducer of CYP3A4 and CYP2C9 | Concentrations of apixaban may decrease  **⭡ risk of thrombosis** |  | **Strong interaction:**  **AVOID if possible**  Consider alternative DOAC (dabigatran, edoxaban, rivaroxaban). If coadministration is unavoidable, monitor closely for apixaban anti-Xa activity, if available. |
| ***Imatinib***  ***Nilotinib***  ***Lapatinib*** | Moderate inhibitors of the CYP3A4 | Possible increased apixaban concentrations  **⭡ risk of bleeding** | None | **Moderate interaction:**  **CAUTION**  Monitor closely for signs of bleeding and measure anti-Xa activity, if necessary. |
| ***Rivaroxaban plus…*** | Partly metabolized by CYP3A4,2J2, liver hydrolytic enzymes. Substrate of P-gp and BCRP. |  | | |
| ***Enzalutamide*** | Strong inducer of CYP3A4; moderate inducer of CYP2C9 and 2C19; weak inducer of CYP1A2 | Concentrations of rivaroxaban may significantly decrease.  **⭡ risk of thrombosis** | Unlikely | **Strong interaction:**  **AVOID**  Consider an alternative DOAC  (edoxaban) or LMWHs. If coadministration is unavoidable, monitor closely for anti-Xa activity, if available. |
| ***Sotorasib*** | Time-dependent  inhibitor of CYP3A; inducer of CYP3A; inhibitor of P-gp. | Plausible mechanisms raise concern over a potentially strong interaction, but there is lack of direct evidence. Thus, the final combined effect of on rivaroxaban exposure is unknown. High variability in response | Not reported. Unlikely | **Moderate to strong interaction:**  **AVOID if possible**  Consider LMWH instead. If coadministration is unavoidable, monitor closely for edoxaban toxicity and efficacy and anti-Xa activity, if available. |
|  |  |  |  |  |
| ***Imatinib***  ***Nilotinib***  ***Lapatinib*** | Moderate inhibitors of CYP3A4 | Possible increased rivaroxaban concentrations.  **⭡ risk of bleeding** | None | **Moderate interaction:**  **CAUTION**  Monitor closely for signs of bleeding and measure anti-Xa activity, if necessary. |
| ***Idelalisib*** | Primary metabolite of idelalisib (GS-563117): strong inhibitor of CYP3A4 | May increase concentrations of apixaban  **⭡ risk of bleeding** | None | **Moderate interaction:**  **CAUTION**  If coadministration is necessary, monitor closely anti-Xa activity, if available. |
| ***Ribociclib*** | Moderate inhibitor of CYP3A4, P-gp and BCRP | Concentrations of rivaroxaban may be increased. High variability in response  **⭡ risk of bleeding** | None | **Moderate interaction:**  **CAUTION**  If coadministration is necessary, monitor closely for apixaban anti-Xa activity, if available. |
| **ALL ANTITHROMBOTIC DRUGS *(antiplatelet and anticoagulant, oral or parenteral drugs) plus …*** | | | | |
| ***Ponatinib, ipililumab, trametinib, ibrutinib, cobimetinib,***  ***nirapanib***  ***tivozanib***  ***acalabrutinib***  ***trastuzumab-emtansine*** | Not CYP-based interaction.  Pharmacodynamic interaction based on an anti-haemostatic effect of each anti-cancer drug alone | **⭡ risk of bleeding** | None | **Clinically relevant interaction:**  **CAUTION**  **AVOID in grade 3-4 TP.**  Closely monitor for signs and symptoms of bleeding. Measure laboratory biomarkers, if available to maintain antithrombotic drugs in the therapeutic range. |

Antithrombotic drugs that may generate clinically relevant DDI with drugs currently used in cancer patients. The possible mechanisms of DDIs are specified, the effect on the safety or efficacy of the antiplatelet drug and consensus advice are provided based on available evidence and/or underlying mechanisms, independent of TP §.

The table is divided into sections according to the type of antithrombotic medication. For each type of antithrombotic medication, only the clinically relevant interactions with drugs used in the cancer setting, are shown. For each antithrombotic medication, concomitant anti-cancer drugs which warrant caution are shaded in orange, while drugs which should be avoided or are contraindicated are shaded in dark red. The summary and advice are based upon data from <https://cancer-druginteractions.org/checker> ^208^

† For each drug, the specific mechanisms of action that are relevant to the DDI in question, are shown.

‡ Hemostatic clinical effects are summarized, and the net effect on bleeding or thrombotic risk (if any) is shown in bold type.

§ The advice refers generically to cancer patients independently of TP, unless otherwise specified.

*AUC*, area under the curve; *BCRP*, breast cancer resistance protein; *CYP*, cytochrome-P; *DAPT*, dual antiplatelet therapy; *DDI*, drug-drug interactions; *DOAC*, direct oral anticoagulant; *ECT*, echarin clotting time; *dTT*, dilute thrombin time; *LMWH*, low molecular weight heparin; *P-gp*, P-glycoprotein; *SAPT,* single antiplatelet therapy; *TP*, thrombocytopenia
